# Two Types of Geometric Jensen–Shannon Divergences

**DOI:** 10.3390/e27090947

**Published:** 2025-09-11

**Authors:** Frank Nielsen

**Affiliations:** Sony Computer Science Laboratories, Tokyo 141-0022, Japan; frank.nielsen.x@gmail.com

**Keywords:** Jensen–Shannon divergence, quasi-arithmetic means, total variation distance, Bhattacharyya distance, Chernoff information, Jeffreys divergence, Taneja divergence, geometric mixtures, exponential families, projective *γ*-divergences, *f*-divergence, separable divergence, information monotonicity

## Abstract

The geometric Jensen–Shannon divergence (G-JSD) has gained popularity in machine learning and information sciences thanks to its closed-form expression between Gaussian distributions. In this work, we introduce an alternative definition of the geometric Jensen–Shannon divergence tailored to positive densities which does not normalize geometric mixtures. This novel divergence is termed the extended G-JSD, as it applies to the more general case of positive measures. We explicitly report the gap between the extended G-JSD and the G-JSD when considering probability densities, and show how to express the G-JSD and extended G-JSD using the Jeffreys divergence and the Bhattacharyya distance or Bhattacharyya coefficient. The extended G-JSD is proven to be an *f*-divergence, which is a separable divergence satisfying information monotonicity and invariance in information geometry. We derive a corresponding closed-form formula for the two types of G-JSDs when considering the case of multivariate Gaussian distributions that is often met in applications. We consider Monte Carlo stochastic estimations and approximations of the two types of G-JSD using the projective γ-divergences. Although the square root of the JSD yields a metric distance, we show that this is no longer the case for the two types of G-JSD. Finally, we explain how these two types of geometric JSDs can be interpreted as regularizations of the ordinary JSD.

## 1. Introduction

### 1.1. Kullback–Leibler and Jensen–Shannon Divergences

Let (X,E,μ) be a measure space on the sample space X, σ-algebra of events E, with μ a prescribed positive measure on the measurable space (X,E) (e.g., counting measure or Lebesgue measure). Let $M_{+}\left(X\right)=\left\{Q\right\}$ be the set of positive distributions *Q* and $M_{+}^{1}\left(X\right)=\left\{P\right\}$ be the subset of probability measures *P*. We denote by $M_{\mu }=\left\{\frac{dQ}{d\mu }\;:\;Q\in M_{+}\left(X\right)\right\}$ and $M_{\mu }^{1}=\left\{\frac{dP}{d\mu }\;:\;P\in M_{+}^{1}\left(X\right)\right\}$ the corresponding sets of Radon–Nikodym positive and probability densities, respectively.

Consider two probability measures $P_{1}$ and $P_{2}$ of $M_{+}^{1}\left(X\right)$ with Radon–Nikodym densities with respect to μ $p_{1}:=\frac{dP_{1}}{d\mu }\in M_{\mu }^{1}$ and $p_{2}:=\frac{dP_{2}}{d\mu }\in M_{\mu }^{1}$, respectively. The deviation of $P_{1}$ to $P_{2}$ (also called distortion, dissimilarity, or deviance) is commonly measured in information theory [1] by the Kullback–Leibler divergence (KLD):

(1) $$\mathrm{KL}\left(p_{1},p_{2}\right):=\int p_{1}\log\frac{p_{1}}{p_{2}}\;d\mu =E_{p_{1}}\left[\log\frac{p_{1}}{p_{2}}\right].$$

Informally, the KLD quantifies the information lost when $p_{2}$ is used to approximate $p_{1}$ by measuring, on average, the surprise when outcomes sampled from $p_{1}$ are assumed to emanate from $p_{2}$: Shannon entropy $H\left(p\right)=\int p\log\frac{1}{p}\;d\mu$ is the expected surprise $H\left(p\right)=E_{p}\left[-\log p\right]$, where −logp(x) measures the surprise of the outcome *x*. Logarithms are taken to base 2 when information is measured in bits, and to base *e* when it is measured in nats. Gibbs’ inequality asserts that $\mathrm{KL}(P_{1},P_{2})\geq 0$ with equality if and only if $P_{1}=P_{2}$ μ-almost everywhere. Since $\mathrm{KL}\left(p_{1},p_{2}\right)\neq \mathrm{KL}\left(p_{2},p_{1}\right)$, various symmetrization schemes of the KLD have been proposed in the literature [1] (e.g., Jeffreys divergence [1,2], resistor average divergence [3] (harmonic KLD symmetrization), Chernoff information [1], etc.)

An important symmetrization technique of the KLD is the Jensen–Shannon divergence [4,5] (JSD):

(2) $$\mathrm{JS}\left(p_{1},p_{2}\right):=\frac{1}{2}\;\left(\mathrm{KL}\left(p_{1},a\right)+\mathrm{KL}\left(p_{2},a\right)\right),$$

where $a=\frac{1}{2}p_{1}+\frac{1}{2}p_{2}$ denotes the statistical mixture of $p_{1}$ and $p_{2}$. The JSD is guaranteed to be upper-bounded by log2 even when the support of $p_{1}$ and $p_{2}$ differ, making it attractive in applications. Furthermore, its square root $\sqrt{\mathrm{JS}}$ yields a metric distance [6,7].

The JSD can be extended to a set of densities to measure the diversity of the set as an information radius [8]. In information theory, the JSD can also be interpreted as an information gain [6] since it can be equivalently written as

$$\mathrm{JS}\left(p_{1},p_{2}\right)=H\left(\frac{1}{2}p_{1}+\frac{1}{2}p_{2}\right)-\frac{H\left(p_{1}\right)+H\left(p_{2}\right)}{2},$$

where H(p)=−∫plogpdμ is Shannon entropy (Shannon entropy for discrete measures and differential entropy for continuous measures). The JSD has also been defined in the setting of quantum information [9], where it has also been proven that its square root yields a metric distance [10].

**Remark** **1.**  *Both the KLD and the JSD belong to the family of f-divergences [11,12] defined for a convex generator f(u) (strictly convex at *1*) by*

$$I_{f}\left(p_{1},p_{2}\right):=\int p_{1}\;f\left(\frac{p_{2}}{p_{1}}\right)\;d\mu .$$

*Indeed, we have $\mathrm{KL}\left(p_{1},p_{2}\right)=I_{f_{\mathrm{KL}}}\left(p_{1},p_{2}\right)$ and $\mathrm{JS}\left(p_{1},p_{2}\right)=I_{f_{\mathrm{JS}}}\left(p_{1},p_{2}\right)$ for the following generators:*

$$\begin{array}{ccc} f_{\mathrm{KL}}\left(u\right) & := & -\log u,\\ f_{\mathrm{JS}}\left(u\right) & := & -\left(1+u\right)\log\frac{1+u}{2}+u\log u. \end{array}$$

*The family of f-divergences is the invariant divergences in information geometry [13]. The f-divergences guarantee information monotonicity by coarse graining [13] (also called lumping in information theory [14]). Using Jensen inequality, we get $I_{f}\left(p_{1},p_{2}\right)\geq f\left(1\right)$.*

**Remark** **2.**  *The metrization of f-divergences was studied in [15]. Once a metric distance $D(p_{1},p_{2})$ is given, we may use the following metric transform [16] to obtain another metric which is guaranteed to be bounded by *1*:*

$$0\leq d\left(p_{1},p_{2}\right)=\frac{D(p_{1},p_{2})}{1+D(p_{1},p_{2})}\leq 1.$$

### 1.2. Jensen–Shannon Symmetrization of Dissimilarities with Generalized Mixtures

In [17], a generalization of the KLD Jensen–Shannon symmetrization scheme was studied for arbitrary statistical dissimilarity D(·,·) using an arbitrary weighted mean [18] $M_{\alpha }$. A generic weighted mean $M_{\alpha }\left(a,b\right)=M_{1-\alpha }\left(b,a\right)$ for $a,b\in R_{>0}$ is a continuous symmetric monotonic map $\alpha \in \left[0,1\right]\mapsto M_{\alpha }\left(a,b\right)$ such that $M_{0}\left(a,b\right)=b$ and $M_{1}\left(a,b\right)=1$. For example, the quasi-arithmetic means [18] are defined according to a monotonous continuous function ϕ as follows:

$$M_{\alpha }^{\phi }(a,b):=\phi^{-1}\left(\alpha \phi (a)+(1-\alpha )\phi (b)\right).$$

When $\phi_{p}\left(u\right)=u^{p}$, we get the *p*-power mean $M_{\alpha }^{\phi_{p}}\left(a,b\right)={\left(\alpha a^{p}+\left(1-\alpha \right)b^{p}\right)}^{\frac{1}{p}}$ for p∈R∖{0}. We extend $\phi_{p}$ for p=0 by defining $\phi_{0}\left(u\right)=\log u$, and get $M_{\alpha }^{\phi_{0}}\left(a,b\right)=a^{\alpha }b^{1-\alpha }$, the weighted geometric mean $G_{\alpha }$.

Let us recall the generalization of the Jensen–Shannon symmetrization scheme of a dissimilarity measure presented in [17]:

**Definition** **1**  ((α,β) M-JS dissimilarity [17])**.** *The Jensen–Shannon skew symmetrization of a statistical dissimilarity D(·,·) with respect to an arbitrary weighted bivariate mean $M_{\alpha }\left(\cdot ,\cdot \right)$ is given by*

(3) $$D_{M_{\alpha },\beta }^{\mathrm{JS}}\left(p_{1},p_{2}\right):=\beta \;D\left(p_{1},{\left(p_{1}p_{2}\right)}_{M_{\alpha }}\right)+\left(1-\beta \right)\;D\left(p_{2},{\left(p_{1}p_{2}\right)}_{M_{\alpha }}\right),\;\left(\alpha ,\beta \right)\in {\left(0,1\right)}^{2},$$

*where ${\left(p_{1}p_{2}\right)}_{M_{\alpha }}$ is the statistical normalized weighted M-mixture of $p_{1}$ and $p_{2}$:*

(4) $${\left(p_{1}p_{2}\right)}_{M_{\alpha }}\left(x\right):=\frac{M_{\alpha }\left(p_{1}\left(x\right),p_{2}\left(x\right)\right)}{\int M_{\alpha }\left(p_{1}\left(x\right),p_{2}\left(x\right)\right)\;d\mu \left(x\right)}.$$

**Remark** **3.**  *A more general definition is given in [17] by using another arbitrary weighted mean $N_{\beta }$ to average the two dissimilarities in Equation *(3)*:*

(5) $$D_{M_{\alpha },N_{\beta }}^{\mathrm{JS}}\left(p_{1},p_{2}\right):=N_{\beta }\left(D\left(p_{1},{\left(p_{1}p_{2}\right)}_{M_{\alpha }}\right),D\left(p_{2},{\left(p_{1}p_{2}\right)}_{M_{\alpha }}\right)\right),\;\left(\alpha ,\beta \right)\in {\left(0,1\right)}^{2}.$$

*When $N_{\beta }=A_{\alpha }$, the weighted arithmetic mean $A_{\alpha }\left(a,b\right)=\alpha a+\left(1-\alpha \right)b$, Equation *(5)* amounts to Equation *(3)*.*

When $\alpha =\frac{1}{2}$, we write, for short, ${\left(p_{1}p_{2}\right)}_{M}$ instead of ${\left(p_{1}p_{2}\right)}_{M_{\frac{1}{2}}}$ in the reminder.

When D=KL, $M=N=A_{\frac{1}{2}}$, Equation (5) yields the Jensen–Shannon divergence of Equation (2): $\mathrm{JS}\left(p_{1},p_{2}\right)={\mathrm{KL}}_{A_{\frac{1}{2}},A_{\frac{1}{2}}}^{\mathrm{JS}}\left(p_{1},p_{2}\right)={\mathrm{KL}}_{A,A}^{\mathrm{JS}}\left(p_{1},p_{2}\right)$.

Lower and upper bounds for the skewed α-Jensen–Shannon divergence were reported in [19].

The abstract mixture normalizer of ${\left(p_{1}p_{2}\right)}_{M_{\alpha }}$ shall be denoted by

$$Z_{M_{\alpha }}\left(p_{1},p_{2}\right):=\int M_{\alpha }\left(p_{1}\left(x\right),p_{2}\left(x\right)\right)\;d\mu \left(x\right),$$

so that the normalized *M*-mixture is written as ${\left(p_{1}p_{2}\right)}_{M_{\alpha }}\left(x\right)=\frac{M_{\alpha }\left(p_{1}\left(x\right),p_{2}\left(x\right)\right)}{Z_{M_{\alpha }}\left(p_{1},p_{2}\right)}$. The normalizer $Z_{M_{\alpha }}\left(p_{1},p_{2}\right)$ is always finite, and thus the weighted *M*-mixtures ${\left(p_{1}p_{2}\right)}_{M_{\alpha }}$ are well-defined:

**Proposition** **1.**  *For any generic weighted mean $M_{\alpha }$, we have the normalizer of the weighted M-mixture bounded by *2*:*

$$0\leq Z_{M_{\alpha }}\left(p_{1},p_{2}\right)\leq 2.$$

**Proof.**  Since $M_{\alpha }$ is a scalar weighted mean, it satisfies the following in-betweenness property:

(6) $$\min\{p_{1}\left(x\right),p_{2}\left(x\right)\}\leq M_{\alpha }\left(p_{1}\left(x\right),p_{2}\left(x\right)\right)\leq \max\{p_{1}\left(x\right),p_{2}\left(x\right)\}.$$

Hence, by using the following two identities for a≥0 and b≥0,

$$\begin{array}{ccc} \min\{a,b\} & = & \frac{a+b}{2}-\frac{1}{2}\left|a-b\right|,\\ \max\{a,b\} & = & \frac{a+b}{2}+\frac{1}{2}\left|a-b\right|, \end{array}$$

we get

(7) $$\begin{array}{ccc} \int \min\{p_{1}\left(x\right),p_{2}\left(x\right)\}\;d\mu \left(x\right)\leq & \int M_{\alpha }\left(p_{1}\left(x\right),p_{2}\left(x\right)\right)\;d\mu \left(x\right) & \leq \int \max\{p_{1}\left(x\right),p_{2}\left(x\right)\}\;d\mu \left(x\right),\\ 0\leq 1-\mathrm{TV}(p_{1},p_{2})\leq & Z_{M_{\alpha }}\left(p_{1},p_{2}\right) & \leq 1+\mathrm{TV}(p_{1},p_{2})\leq 2, \end{array}$$

where

$$\mathrm{TV}\left(p_{1},p_{2}\right):=\frac{1}{2}\int \left|p_{1}-p_{2}\right|\;d\mu ,$$

is the total variation distance, upper-bounded by 1. When the support of the densities $p_{1}$ and $p_{2}$ intersect (i.e., non-singular probability measures $P_{1}$ and $P_{2}$), we have $Z_{M_{\alpha }}\left(p_{1},p_{2}\right)>0$, and therefore the weighted *M*-mixtures ${\left(p_{1}p_{2}\right)}_{M_{\alpha }}$ are well-defined. □

The generic Jensen–Shannon symmetrization of dissimilarities given in Definition 1 allows us to re-interpret some well-known statistical dissimilarities:

For example, the Chernoff information [1,20] is defined by

(8) $$C\left(p_{1},p_{2}\right):=\max_{\alpha \in (0,1)}B_{\alpha }\left(p_{1},p_{2}\right),$$

where $B_{\alpha }\left(p_{1},p_{2}\right)$ denotes the α-skewed Bhattacharrya distance:

(9) $$B_{\alpha }\left(p_{1},p_{2}\right):=-\log\int p_{1}^{\alpha }\;p_{2}^{1-\alpha }\;d\mu$$

When $\alpha =\frac{1}{2}$, we note $B\left(p_{1},p_{2}\right)=B_{\frac{1}{2}}\left(p_{1},p_{2}\right)$, the Bhattacharrya distance. Notice that the Bhattacharrya distance is not a metric distance as it violates the triangle inequality of metrics.

Using the framework of JS-symmetrization of dissimilarities, we can reinterpret the Chernoff information as

$$C\left(p_{1},p_{2}\right)={\left({\mathrm{KL}}^{*}\right)}_{G_{\alpha^{*}},A_{\frac{1}{2}}}^{\mathrm{JS}}\left(p_{1},p_{2}\right),$$

where $\alpha^{*}$ is provably the unique optimal skewing factor in Equation (8), such that we have [20]:

$$\begin{array}{ccc} C(p_{1},p_{2}) & = & {\mathrm{KL}}^{*}\left(p_{1},{\left(p_{1}p_{2}\right)}_{G_{\alpha^{*}}}\right)={\mathrm{KL}}^{*}\left(p_{2},{\left(p_{1}p_{2}\right)}_{G_{\alpha^{*}}}\right),\\ & = & \frac{1}{2}\;\left({\mathrm{KL}}^{*}\left(p_{1},{\left(p_{1}p_{2}\right)}_{G_{\alpha^{*}}}\right)+{\mathrm{KL}}^{*}\left(p_{2},{\left(p_{1}p_{2}\right)}_{G_{\alpha^{*}}}\right)\right), \end{array}$$

where ${\mathrm{KL}}^{*}$ denotes the reverse KLD:

$${\mathrm{KL}}^{*}\left(p_{1},p_{2}\right):=\mathrm{KL}\left(p_{2},p_{1}\right).$$

Note that the KLD is sometimes called the forward KLD (e.g., [21]), and we have ${{\mathrm{KL}}^{*}}^{*}\left(p_{1},p_{2}\right)=\mathrm{KL}\left(p_{1},p_{2}\right)$.

Although arithmetic mixtures are most often used in statistics, geometric mixtures are also encountered, for example in Bayesian statistics [22] or in Markov chain Monte Carlo annealing [23], just to give two examples. In information geometry, statistical power mixtures based on the homogeneous power means are used to perform stochastic integration of statistical models [24].

**Proposition** **2** (Bhattacharyya distance as G-JSD)**.** *The Bhattacharyya distance [25] and the α-skewed Bhattacharyya distances can be interpreted as JS-symmetrizations of the reverse KLD with respect to the geometric mean G:*

$$\begin{array}{ccc} B(p_{1},p_{2}) & := & -\log\int \sqrt{p_{1}\;p_{2}}\;d\mu ={\left({\mathrm{KL}}^{*}\right)}_{G}^{\mathrm{JS}}\left(p_{1},p_{2}\right),\\ B_{\alpha }\left(p_{1},p_{2}\right) & := & -\log\int p_{1}^{\alpha }\;p_{2}^{1-\alpha }\;d\mu ={\left({\mathrm{KL}}^{*}\right)}_{G_{\alpha }}^{\mathrm{JS}}\left(p_{1},p_{2}\right). \end{array}$$

**Proof.**  Let $m={\left(p_{1}p_{2}\right)}_{G}=\frac{\sqrt{p_{1}p_{2}}}{Z(p_{1},p_{2})}$ denote the weighted geometric mixture with the normalizer $Z_{G}\left(p_{1},p_{2}\right)=\int \sqrt{p_{1}p_{2}}\;d\mu$. By definition of the JS-symmetrization of the reverse KLD, we have

$$\begin{array}{ccc} {\left({\mathrm{KL}}^{*}\right)}_{G}^{\mathrm{JS}}\left(p_{1},p_{2}\right) & := & \frac{1}{2}\left({\mathrm{KL}}^{*}\left(p_{1},{\left(p_{1}p_{2}\right)}_{G}\right)+{\mathrm{KL}}^{*}\left(p_{2},{\left(p_{1}p_{2}\right)}_{G}\right)\right),\\ & = & \frac{1}{2}\left(\mathrm{KL}\left({\left(p_{1}p_{2}\right)}_{G},p_{1}\right)+\mathrm{KL}\left({\left(p_{1}p_{2}\right)}_{G},p_{2}\right)\right),\\ & = & \frac{1}{2}\left(\int \left(m\;\log\frac{\sqrt{p_{1}p_{2}}}{p_{1}\;Z_{G}\left(p_{1},p_{2}\right)}+m\;\log\frac{\sqrt{p_{1}p_{2}}}{p_{2}\;Z_{G}\left(p_{1},p_{2}\right)}\right)\;d\mu \right),\\ & = & \frac{1}{2}\left(\int \frac{1}{2}m\log\frac{p_{2}}{p_{1}}\;\frac{p_{1}}{p_{2}}d\mu -2\log Z_{G}\left(p_{1},p_{2}\right)\int m\;d\mu \right),\\ & = & -\log Z_{G}\left(p_{1},p_{2}\right)=:B\left(p_{1},p_{2}\right). \end{array}$$

The proof carries on similarly for the α-skewed JS-symmetrization of the reverse KLD: we now let $m_{\alpha }={\left(p_{1}p_{2}\right)}_{G_{\alpha }}=\frac{p_{1}^{\alpha }p_{2}^{1-\alpha }}{Z_{G_{\alpha }}\left(p_{1},p_{2}\right)}$ be the α-weighted geometric mixture with the normalizer $Z_{G_{\alpha }}\left(p_{1},p_{2}\right)=\int p_{1}^{\alpha }p_{2}^{1-\alpha }\;d\mu$, written as $Z_{G_{\alpha }}$ for short below:

$$\begin{array}{ccc} {{\mathrm{KL}}^{*}}_{G_{\alpha },\alpha }^{\mathrm{JS}}\left(p_{1},p_{2}\right) & := & \alpha \;{\mathrm{KL}}^{*}\left(p_{1},{\left(p_{1}p_{2}\right)}_{G_{\alpha }}\right)+\left(1-\alpha \right)\;{\mathrm{KL}}^{*}\left(p_{2},{\left(p_{1}p_{2}\right)}_{G_{\alpha }}\right),\\ & = & \alpha \;\mathrm{KL}\left(m_{\alpha },p_{1}\right)+\left(1-\alpha \right)\;\mathrm{KL}\left(m_{\alpha },p_{2}\right),\\ & = & \int \left(\alpha m_{\alpha }\log\frac{p_{1}^{\alpha }\;p_{2}^{1-\alpha }}{Z_{G_{\alpha }}\;p_{1}}+\left(1-\alpha \right)m_{\alpha }\log\frac{p_{1}^{\alpha }p_{2}^{1-\alpha }}{Z_{G_{\alpha }}\;p_{2}}\right)d\mu ,\\ & = & -\left(\alpha +1-\alpha \right)\log Z_{G_{\alpha }}\int m_{\alpha }\;d\mu +\int m_{\alpha }\log{\left(\frac{p_{2}}{p_{1}}\right)}^{\alpha (1-\alpha )}\;{\left(\frac{p_{1}}{p_{2}}\right)}^{\alpha (1-\alpha )}d\mu ,\\ & = & -\log Z_{G_{\alpha }}\left(p_{1},p_{2}\right)=:B_{\alpha }\left(p_{1},p_{2}\right). \end{array}$$

□

Besides information theory [1], the JSD also plays an important role in machine learning [26,27,28]. However, one drawback that refrains its use in practice is that the JSD between two Gaussian distributions (normal distributions) is not known in closed form, since no analytic formula is known for the differential entropy of a two-component Gaussian mixture [29], and thus the JSD needs to be numerically approximated in practice by various methods.

To circumvent this problem, the geometric G-JSD was defined in [17] as follows:

**Definition** **2**  (G-JSD [17])**.** *The geometric Jensen–Shannon divergence (G-JSD) between two probability densities, $p_{1}$ and $p_{2}$, is defined by*

$${\mathrm{JS}}_{G}\left(p_{1},p_{2}\right):=\frac{1}{2}\;\left(\mathrm{KL}\left(p_{1},{\left(p_{1}p_{2}\right)}_{G}\right)+\mathrm{KL}\left(p_{2},{\left(p_{1}p_{2}\right)}_{G}\right)\right),$$

*where ${\left(p_{1}p_{2}\right)}_{G}\left(x\right)=\frac{\sqrt{p_{1}\left(x\right)\;p_{2}\left(x\right)}}{\int \sqrt{p_{1}\left(x\right)\;p_{2}\left(x\right)}\;d\mu }$ is the (normalized) geometric mixture of $p_{1}$ and $p_{2}$.*

We have ${\mathrm{JS}}_{G}\left(p_{1},p_{2}\right)={\mathrm{KL}}_{G}^{\mathrm{JS}}\left(p_{1},p_{2}\right)$. Since, by default, the *M*- mixture JS-symmetrization of dissimilarities *D* is performed on the right argument (i.e., $D_{M}^{\mathrm{JS}}$), we may also consider a dual JS-symmetrization by setting the *M*-mixtures on the left argument. We denote this left mixture JS-symmetrization with $D_{M}^{{\mathrm{JS}}^{*}}$. We have $D_{M}^{{\mathrm{JS}}^{*}}\left(p_{1},p_{2}\right)={\left(D^{*}\right)}_{M}^{\mathrm{JS}}\left(p_{1},p_{2}\right)$, i.e., the left-sided JS-symmetrization of *D* amounts to a right-sided JS-symmetrization of the dual dissimilarity $D^{*}\left(p_{1},p_{2}\right):=D\left(p_{2},p_{1}\right)$.

Thus, a left-sided G-JSD divergence ${\mathrm{JS}}_{G}^{*}$ was also defined in [17]:

**Definition** **3.**  *The left-sided geometric Jensen–Shannon divergence (G-JSD) between two probability densities $p_{1}$ and $p_{2}$ is defined by*

$$\begin{array}{ccc} {\mathrm{JS}}_{G}^{*}\left(p_{1},p_{2}\right) & := & \frac{1}{2}\;\left(\mathrm{KL}\left({\left(p_{1}p_{2}\right)}_{G},p_{1}\right)+\mathrm{KL}\left({\left(p_{1}p_{2}\right)}_{G},p_{2}\right)\right),\\ & = & \frac{1}{2}\;\left({\mathrm{KL}}^{*}\left(p_{1},{\left(p_{1}p_{2}\right)}_{G}\right)+{\mathrm{KL}}^{*}\left(p_{2},{\left(p_{1}p_{2}\right)}_{G}\right)\right), \end{array}$$

*where ${\left(p_{1}p_{2}\right)}_{G}\left(x\right)=\frac{\sqrt{p_{1}\left(x\right)\;p_{2}\left(x\right)}}{\int \sqrt{p_{1}\left(x\right)\;p_{2}\left(x\right)}\;d\mu }$ is the (normalized) geometric mixture of $p_{1}$ and $p_{2}$.*

To contrast with the numerical approximation limitation of the JSD between Gaussians, one advantage of the geometric Jensen–Shannon divergence (G-JSD) is that it admits a closed-form expression between Gaussian distributions [17]. However, the G-JSD is no longer bounded. The G-JSD formula between Gaussian distributions has been used in several scenarios. See [30,31,32,33,34,35,36,37,38] for a few use cases.

Let us express the G-JSD divergence using other familiar divergences.

**Proposition** **3.**  *We have the following expression of the geometric Jensen–Shannon divergence:*

$${\mathrm{JS}}_{G}\left(p_{1},p_{2}\right)=\frac{1}{4}\;J\left(p_{1},p_{2}\right)-B\left(p_{1},p_{2}\right),$$

*where $J\left(p_{1},p_{2}\right):=\int \left(p_{1}-p_{2}\right)\log\frac{p_{1}}{p_{2}}\;d\mu$ is Jeffreys’ divergence [2], and*

$$B\left(p_{1},p_{2}\right)=-\log\int \sqrt{p_{1}p_{2}}\;d\mu =-\log Z_{G}\left(p_{1},p_{2}\right),$$

*is the Bhattacharrya distance.*

**Proof.**  We have the following:

$$\begin{array}{ccc} {\mathrm{JS}}_{G}\left(p_{1},p_{2}\right) & := & \frac{1}{2}\left(\mathrm{KL}\left(p_{1},{\left(p_{1}p_{2}\right)}_{G}\right)+\mathrm{KL}\left(p_{2},{\left(p_{1}p_{2}\right)}_{G}\right)\right),\\ & = & \frac{1}{2}\left(\int \left(p_{1}\left(x\right)\log\frac{p_{1}\left(x\right)\;Z_{G}\left(p_{1},p_{2}\right)}{\sqrt{p_{1}\left(x\right)\;p_{2}\left(x\right)}}+p_{2}\left(x\right)\log\frac{p_{2}\left(x\right)\;Z_{G}\left(p_{1},p_{2}\right)}{\sqrt{p_{1}\left(x\right)\;p_{2}\left(x\right)}}\right)d\mu \left(x\right)\right),\\ & = & \frac{1}{2}\left(\int \left(p_{1}\left(x\right)+p_{2}\left(x\right)\right)\log Z_{G}\left(p_{1},p_{2}\right)\;d\mu \left(x\right)+\frac{1}{2}\mathrm{KL}\left(p_{1},p_{2}\right)+\frac{1}{2}\mathrm{KL}\left(p_{2},p_{1}\right)\right),\\ & = & \log Z_{G}\left(p_{1},p_{2}\right)+\frac{1}{4}J\left(p_{1},p_{2}\right),\\ & = & \frac{1}{4}J\left(p_{1},p_{2}\right)-B\left(p_{1},p_{2}\right). \end{array}$$

□

**Corollary** **1** (G-JSD upper bound)**.** *We have the upper bound ${\mathrm{JS}}_{G}\left(p,q\right)\leq \frac{1}{4}\;J\left(p,q\right)$.*

**Proof.**  Since $B(p_{1},p_{2})\geq 0$ and ${\mathrm{JS}}_{G}\left(p_{1},p_{2}\right)=\frac{1}{4}\;J\left(p_{1},p_{2}\right)-B\left(p_{1},p_{2}\right)$, we have ${\mathrm{JS}}_{G}\left(p,q\right)\leq \frac{1}{4}\;J\left(p,q\right)$. □

**Remark** **4.**  *Although the KLD and JSD are separable divergences (i.e., f-divergences expressed as integrals of scalar divergences), the M-JSD divergence is, in general, not separable, because it requires mixtures to be normalized inside the log terms. Notice that the Bhattacharyya distance is, similarly, not a separable divergence, but the Bhattacharyya similarity coefficient $\mathrm{BC}\left(p_{1},p_{2}\right)=\exp\left(-B\left(p_{1},p_{2}\right)\right)=\int \sqrt{p_{1}\;p_{2}}\;d\mu$ is a separable “f-divergence”/f-coefficient for $f_{\mathrm{BC}}\left(u\right)=\sqrt{u}$ (here, a concave generator): $\mathrm{BC}\left(p_{1},p_{2}\right)=I_{f_{\mathrm{BC}}}\left(p_{1},p_{2}\right)$. Notice that $f_{\mathrm{BC}}\left(1\right)=1$, and because of the concavity of $f_{\mathrm{BC}}$, we have $I_{f_{\mathrm{BC}}}\left(p_{1},p_{2}\right)\leq f_{\mathrm{BC}}\left(1\right)=1$ (hence, the term f-coefficient to reflect the notion of a similarity measure).*

### 1.3. Paper Outline

The paper is organized as follows: We first give an alternative definition of the M-JSD in Section 2 (Definition 4) which extends to positive measures and does not require normalization of geometric mixtures. We call this new divergence the extended M-JSD, and we compare the two types of geometric JSDs when dealing with probability measures. In Section 4, we show that these normalized/extended *M*-JSD divergences can be interpreted as regularizations of the Jensen–Shannon divergence, and exhibit several bounds. We discuss Monte Carlo stochastic approximations and approximations using γ-divergences [39] in Section 5. For the case of geometric mixtures, although the G-JSD is not an *f*-divergence, we show that the extended G-JSD is an *f*-divergence (Proposition 5), and we express both the G-JSD and the extended G-JSD using both the Jeffreys divergence and the Bhattacharyya divergence or coefficient. We report a related closed-form formula for the G-JSD and extended G-JSD between two Gaussian distributions in Section 3. Finally, we summarize the main results in the concluding section, Section 6.

A list of notations is provided in Nomenclature.

## 2. A Novel Definition: The G-JSD, Extended to Positive Measures

### 2.1. Definition and Properties

We may consider the following two modifications of the G-JSD:

- First, we replace the KLD with the extended KLD between positive densities $q_{1}\in M_{\mu }^{+}$ and $q_{2}\in M_{\mu }^{+}$ instead of normalized densities:
  (10) $${\mathrm{KL}}^{+}\left(q_{1},q_{2}\right):=\int \left(q_{1}\log\frac{q_{1}}{q_{2}}+q_{2}-q_{1}\right)\;d\mu ,$$
  (with ${\mathrm{KL}}^{+}\left(p_{1},p_{2}\right)=\mathrm{KL}\left(p_{1},p_{2}\right)$);
- Second, we consider unnormalized *M*-mixture densities:
  $${\left(q_{1}q_{2}\right)}_{{\tilde{M}}_{\alpha }}\left(x\right):=M_{\alpha }\left(q_{1}\left(x\right),q_{2}\left(x\right)\right),$$
  where we use the $\tilde{M}$ tilde notation to indicate that the *M*-mixture is not normalized, instead of normalized densities ${\left(q_{1}q_{2}\right)}_{M_{\alpha }}\left(x\right)$.

The extended KLD can be interpreted as a pointwise integral of a scalar Bregman divergence obtained for the negative Shannon entropy generator [40]. This proves that ${\mathrm{KL}}^{+}\left(q_{1},q_{2}\right)\geq 0$ with equality if and only if $q_{1}=q_{2}$ μ-almost everywhere. Notice that $\mathrm{KL}(q_{1},q_{2})$ may be negative when $q_{1}$ and/or $q_{2}$ are not normalized to probability densities, but we always have ${\mathrm{KL}}^{+}\left(q_{1},q_{2}\right)\geq 0$.

The extended KLD is an extended *f*-divergence [41]: ${\mathrm{KL}}^{+}\left(q_{1},q_{2}\right)=I_{f_{{\mathrm{KL}}^{+}}}^{+}\left(q_{1},q_{2}\right)$ for $f_{{\mathrm{KL}}^{+}}\left(u\right)=-\log\left(u\right)+u-1$, where $I_{f}^{+}\left(q_{1},q_{2}\right)$ denotes the *f*-divergence extended to positive densities $q_{1}$ and $q_{2}$:

$$I_{f}^{+}\left(q_{1},q_{2}\right)=\int q_{1}\;f\left(\frac{q_{2}}{q_{1}}\right)\;d\mu .$$

**Remark** **5.**  *As a side remark, it is preferable in practice to estimate the KLD between $p_{1}$ and $p_{2}$ by Monte Carlo methods using Equation *(10)* instead of Equation *(1)* in order to guarantee the non-negativeness of the KLD (Gibbs’ inequality). Indeed, the sampling of s samples $x_{1},\ldots ,x_{s}$, defining two unnormalized distributions $q_{1}\left(x\right)=\frac{1}{s}\sum_{i=1}^{s}p_{1}\left(x\right)\delta_{x_{i}}\left(x\right)$ and $q_{2}\left(x\right)=\frac{1}{s}\sum_{i=1}^{s}p_{2}\left(x\right)\delta_{x_{i}}\left(x\right)$, where*

$$\delta_{x_{i}}\left(x\right)=\left\{\begin{array}{cc} 1, & ifx=x_{i}\\ 0, & otherwise \end{array}\right..$$

**Remark** **6.**  *For an arbitrary distortion measure $D^{+}\left(q_{1},q_{2}\right)$ between positive measures $q_{1}$ and $q_{2}$, we can build a corresponding projective divergence $\tilde{D}\left(q_{1},q_{2}\right)$ as follows:*

$$\tilde{D}\left(q_{1},q_{2}\right):=D^{+}\left(\frac{q_{1}}{Z(q_{1})},\frac{q_{1}}{Z(q_{2})}\right),$$

*where Z(q):=∫qdμ is the normalization factor of the positive density q. The divergence $\tilde{D}$ is said to be projective because we have, for all $\lambda_{1}>0,\;\lambda_{2}>0$, the property that $\tilde{D}\left(\lambda_{1}q_{1},\lambda_{2}q_{2}\right)=\tilde{D}\left(q_{1},q_{2}\right)=D^{+}\left(p_{1},p_{2}\right)$, where $p_{i}=\frac{q_{i}}{Z(q_{i})}$ are the normalized densities. The projective Kullback–Leibler divergence $\tilde{\mathrm{KL}}$ is thus another projective extension of the KLD to non-normalized densities which coincide with the KLD for probability densities. But the projective KLD is different from the extended KLD of Equation *(10)*, and furthermore, we have $\tilde{\mathrm{KL}}\left(q_{1},q_{2}\right)=0$ if and only if $q_{1}=\lambda \;q_{2}$ μ-almost everywhere for some λ>0.*

Let us now define the Jensen–Shannon symmetrization of an extended statistical divergence $D^{+}$ with respect to an arbitrary weighted mean $M_{\alpha }$ as follows:

**Definition** **4** (Extended M-JSD)**.** *A Jensen–Shannon skew symmetrization of a statistical divergence $D^{+}\left(\cdot ,\cdot \right)$ between two positive measures $q_{1}$ and $q_{2}$ with respect to a weighted mean $M_{\alpha }$ is defined by*

(11) $$D_{{\tilde{M}}_{\alpha },\beta }^{{\mathrm{JS}}^{+}}\left(q_{1},q_{2}\right):=\beta \;D^{+}\left(q_{1},{\left(q_{1}q_{2}\right)}_{{\tilde{M}}_{\alpha }}\right)+\left(1-\beta \right)\;D^{+}\left(q_{1},{\left(q_{1}q_{2}\right)}_{{\tilde{M}}_{\alpha }}\right),$$

When $\beta =\frac{1}{2}$, we write, for short, $D_{{\tilde{M}}_{\alpha }}^{{\mathrm{JS}}^{+}}\left(q_{1},q_{2}\right)$, and furthermore, when $\alpha =\frac{1}{2}$, we simplify the notation to $D_{\tilde{M}}^{{\mathrm{JS}}^{+}}\left(q_{1},q_{2}\right)$.

When $D^{+}={\mathrm{KL}}^{+}$, we obtain the extended geometric Jensen–Shannon divergence, ${\mathrm{JS}}_{\tilde{G}}^{+}\left(q_{1},q_{2}\right)={\mathrm{KL}}_{\tilde{G}}^{{\mathrm{JS}}^{+}}\left(q_{1},q_{2}\right)$:

**Definition** **5** (Extended G-JSD)**.** *The extended geometric Jensen–Shannon divergence between two positive densities $q_{1}$ and $q_{2}$ is*

(12) $$\begin{array}{ccc} {\mathrm{JS}}_{\tilde{G}}^{+}\left(q_{1},q_{2}\right) & = & \frac{1}{2}\;\left({\mathrm{KL}}^{+}\left(q_{1},{\left(q_{1}q_{2}\right)}_{\tilde{G}}\right)+{\mathrm{KL}}^{+}\left(q_{2},{\left(q_{1}q_{2}\right)}_{\tilde{G}}\right))\right), \end{array}$$

The extended G-JSD between two normalized densities $p_{1}$ and $p_{2}$ is thus

(13) $$\begin{array}{ccc} {\mathrm{JS}}_{\tilde{G}}^{+}\left(p_{1},p_{2}\right) & = & \frac{1}{2}\left(\int \left(p_{1}\log\frac{p_{1}}{\sqrt{p_{1}\;p_{2}}}+p_{2}\log\frac{p_{2}}{\sqrt{p_{1}\;p_{2}}}\right)\;d\mu +\int \sqrt{p_{1}\;p_{2}}\;d\mu )\right)-1, \end{array}$$

(14) $$\begin{array}{ccc} & = & \frac{1}{2}\left(\int \left(p_{1}\log\sqrt{\frac{p_{1}}{p_{2}}}+p_{2}\log\sqrt{\frac{p_{2}}{p_{1}}}\right)\;d\mu +Z_{G}\left(p_{1},p_{2}\right)\right)-1, \end{array}$$

with $Z_{G}\left(p_{1},p_{2}\right)=\exp\left(-B\left(p_{1},p_{2}\right)\right)$.

Thus, we get the following propositions:

**Proposition** **4.**  *The extended geometric Jensen–Shannon divergence (G-JSD) can be expressed as follows:*

$${\mathrm{JS}}_{\tilde{G}}^{+}\left(p_{1},p_{2}\right)=\frac{1}{4}\;J\left(p_{1},p_{2}\right)+\exp\left(-B\left(p_{1},p_{2}\right)\right)-1.$$

**Proof.**  We have

$$\begin{array}{ccc} {\mathrm{JS}}_{\tilde{G}}^{+}\left(p_{1},p_{2}\right) & = & \frac{1}{2}\;\left({\mathrm{KL}}^{+}\left(p_{1},{\left(p_{1}p_{2}\right)}_{\tilde{G}}\right)+{\mathrm{KL}}^{+}\left(p_{2},{\left(p_{1}p_{2}\right)}_{\tilde{G}}\right)\right),\\ & = & \frac{1}{2}\;\left(\int \left(p_{1}\log\sqrt{\frac{p_{1}}{p_{2}}}+p_{2}\log\sqrt{\frac{p_{2}}{p_{1}}}+2\sqrt{p_{1}\;p_{2}}-\left(p_{1}+p_{2}\right)\right)\;d\mu \right),\\ & = & \int \frac{1}{4}\;\left(p_{1}-p_{2}\right)\log\frac{p_{1}}{p_{2}}\;d\mu +\int \sqrt{p_{1}\;p_{2}}\;d\mu -1,\\ & = & \frac{1}{4}\;J\left(p_{1},p_{2}\right)+\exp\left(-B\left(p_{1},p_{2}\right)\right)-1. \end{array}$$

□

Thus, we can express the gap between ${\mathrm{JS}}_{\tilde{G}}^{+}\left(p_{1},p_{2}\right)$ and ${\mathrm{JS}}_{G}\left(p_{1},p_{2}\right)$:

$$\Delta_{G}\left(p_{1},p_{2}\right)={\mathrm{JS}}_{\tilde{G}}^{+}\left(p_{1},p_{2}\right)-{\mathrm{JS}}_{G}\left(p_{1},p_{2}\right)=\exp\left(-B\left(p_{1},p_{2}\right)\right)+B\left(p_{1},p_{2}\right)-1.$$

Since $Z_{G}\left(p_{1},p_{2}\right)=\exp\left(-B\left(p_{1},p_{2}\right)\right)$, we have:

$$\Delta_{G}\left(p_{1},p_{2}\right)=Z_{G}\left(p_{1},p_{2}\right)-\log Z_{G}\left(p_{1},p_{2}\right)-1.$$

**Proposition** **5.**  *The extended G-JSD is an f-divergence for the generator*

$$f_{\tilde{G}}\left(u\right)=\frac{1}{4}\left(u-1\right)\log u+\sqrt{u}-1.$$

*That is, we have ${\mathrm{JS}}_{\tilde{G}}^{+}\left(p_{1},p_{2}\right)=I_{f_{\tilde{G}}}\left(p_{1},p_{2}\right)$.*

**Proof.**  We proved that ${\mathrm{JS}}_{\tilde{G}}^{+}\left(p_{1},p_{2}\right)=\frac{1}{4}\;J\left(p_{1},p_{2}\right)+\mathrm{BC}\left(p_{1},p_{2}\right)-1$. The Jeffreys divergence is an *f*-divergence for the generator $f_{J}\left(u\right)=\left(u-1\right)\log u$, and the Bhattacharyya coefficient is an *f*-coefficient for $f_{\mathrm{BC}}\left(u\right)=\sqrt{u}$ (a “*f*-divergence” for a concave generator). Thus, we have

$$f_{\tilde{G}}\left(u\right)=\frac{1}{4}\left(u-1\right)\log u+\sqrt{u}-1,$$

such that ${\mathrm{JS}}_{\tilde{G}}^{+}\left(p_{1},p_{2}\right)=I_{f_{\tilde{G}}}\left(p_{1},p_{2}\right)$. We check that $f_{\tilde{G}}\left(u\right)$ is convex, since $f_{\tilde{G}}^{\prime \prime }\left(u\right)=\frac{\sqrt{u}\left(u+1\right)-u}{4u^{\frac{5}{2}}}$ (and by a change of variable $t=\sqrt{u}$, the numerator $t(t^{2}-t+1)$ is shown to be positive, since the discriminant of $t^{2}-t+1$ is negative), and we have $f_{\tilde{G}}\left(1\right)=0$. Thus, the extended G-JSD is a proper *f*-divergence. □

It follows that the extended G-JSD satisfies the information monotonicity of invariant divergences in information geometry [13].

By abuse of notations, we have

$${\mathrm{KL}}^{+}\left(q_{1},q_{2}\right):=\mathrm{KL}\left(q_{1},q_{2}\right)+\int \left(q_{2}-q_{1}\right)\;d\mu ,$$

although $q_{1}$ and $q_{2}$ may not need to be normalized in the KL term (which can then yield a potentially negative value). Letting $Z\left(q_{i}\right):=\int q_{i}\;d\mu$ be the total mass of positive density $q_{i}$, we have

(15) $${\mathrm{KL}}^{+}\left(q_{1},q_{2}\right)=\mathrm{KL}\left(q_{1},q_{2}\right)+Z\left(q_{2}\right)-Z\left(q_{1}\right).$$

Let ${\tilde{m}}_{\alpha }=M_{\alpha }\left(q_{1},q_{2}\right)$ be the unnormalized *M*-mixture of positive densities $q_{1}$ and $q_{2}$, and set $Z_{M_{\alpha }}=\int {\tilde{m}}_{\alpha }\;d\mu$ be the normalization term so that we have $m_{\alpha }=\frac{{\tilde{m}}_{\alpha }}{Z_{M_{\alpha }}}$ and ${\tilde{m}}_{\alpha }=Z_{M_{\alpha }}\;m_{\alpha }$. When clear from context, we write $Z_{\alpha }$ instead of $Z_{M_{\alpha }}$.

We get, after elementary calculus, the following identity:

(16) $${\mathrm{JS}}_{{\tilde{M}}_{\alpha },\beta }^{+}\left(q_{1},q_{2}\right)={\mathrm{JS}}_{M_{\alpha },\beta }\left(q_{1},q_{2}\right)-\left(\beta Z\left(q_{1}\right)+\left(1-\beta \right)Z\left(q_{2}\right)\right)\log Z_{\alpha }+Z_{\alpha }-\left(\beta Z\left(q_{1}\right)+\left(1-\beta \right)Z\left(q_{2}\right)\right).$$

Therefore, the difference gap $\Delta_{M_{\alpha },\beta }\left(p_{1},p_{2}\right)$ (written for short as $\Delta (p_{1},p_{2})$) between the normalized JSD and the unnormalized M-JSD between two normalized densities $p_{1}$ and $p_{2}$ (i.e., with $Z_{1}=Z\left(p_{1}\right)=1$ and $Z_{2}=Z\left(p_{2}\right)=1$) is

(17) $$\Delta \left(p_{1},p_{2}\right):={{\mathrm{JS}}^{+}}_{{\tilde{M}}_{\alpha },\beta }\left(p_{1},p_{2}\right)-{\mathrm{JS}}^{M_{\alpha },\beta }\left(p_{1},p_{2}\right)=Z_{\alpha }-\log\left(Z_{\alpha }\right)-1.$$

**Proposition** **6** (Extended/normalized M-JSD Gap)**.** *The following identity holds:*

$${{\mathrm{JS}}^{+}}_{{\tilde{M}}_{\alpha },\beta }\left(p_{1},p_{2}\right)={\mathrm{JS}}_{M_{\alpha },\beta }\left(p_{1},p_{2}\right)+Z_{\alpha }-\log\left(Z_{\alpha }\right)-1.$$

Thus, ${{\mathrm{JS}}^{+}}_{{\tilde{M}}_{\alpha },\beta }\left(p_{1},p_{2}\right)\geq {\mathrm{JS}}_{M_{\alpha },\beta }\left(p_{1},p_{2}\right)$ when $\Delta (p_{1},p_{2})\geq 0$, and ${{\mathrm{JS}}^{+}}_{{\tilde{M}}_{\alpha },\beta }\left(p_{1},p_{2}\right)\leq {\mathrm{JS}}_{M_{\alpha },\beta }\left(p_{1},p_{2}\right)$ when $\Delta (p_{1},p_{2})\leq 0$.

When we consider the weighted arithmetic mean $A_{\alpha }$, we always have $Z_{\alpha }=1$ for α∈(0,1), and thus the two definitions (Definition 1 and Definition 4) of the *A*-JSD coincide (i.e., $Z_{\alpha }^{A}-\log\left(Z_{\alpha }^{A}\right)-1=0$):

$${\mathrm{JS}}_{A}\left(p_{1},p_{2}\right)={\mathrm{JS}}_{\tilde{A}}\left(p_{1},p_{2}\right).$$

However, when the weighted mean $M_{\alpha }$ differs from the weighted arithmetic mean (i.e., $M_{\alpha }\neq A_{\alpha }$), the two definitions of the M-JSD ${\mathrm{JS}}_{M}$ and extended M-JSD ${\mathrm{JS}}_{\tilde{M}}$ differ by the gap expressed in Equation (17).

**Remark** **7.**  *When information is measured in bits, logarithms are taken to base *2*, and when information is measured in nats, base e is considered. Thus, we shall generally consider the gap $\Delta_{b}=Z_{\alpha }-\log_{b}\left(Z_{\alpha }\right)-1$, where b denotes the base of the logarithm. When b=e, we have $\Delta_{e}\geq 0$ for all $Z_{\alpha }>0$. When b=2, we have $\Delta_{2}=Z_{\alpha }-\log_{2}\left(Z_{\alpha }\right)-1\geq 0$ when $0<Z_{\alpha }\leq 1$ or $Z_{\alpha }\geq 2$. But since $Z_{\alpha }\leq 2$ (see Equation *(7)*), the condition simplifies to $\Delta_{2}\geq 0$ if and only if $Z_{\alpha }\leq 1$.*

**Remark** **8.**  *Although $\sqrt{\mathrm{JS}}$ is a metric distance [5], $\sqrt{{\mathrm{JS}}_{G}}$ is not a metric distance, as the triangle inequality is not satisfied. It suffices to report a counterexample of the triangle inequality for a triple of points $p_{1}$, $p_{2}$, and $p_{3}$: Consider $p_{1}=\left(0.55,0.45\right)$, $p_{2}=\left(0.002,0.998\right)$, and $p_{3}=\left(0.045,0.955\right)$. Then we have $\sqrt{{\mathrm{JS}}_{G}}\left(p_{1},p_{2}\right)\approx 1.0263227\ldots$, $\sqrt{{\mathrm{JS}}_{G}}\left(p_{1},p_{3}\right)\approx 0.63852342\ldots$, and $\sqrt{{\mathrm{JS}}_{G}}\left(p_{3},p_{2}\right)\approx 0.19794622\ldots$. The triangle inequality fails with an error of*

$$\sqrt{{\mathrm{JS}}_{G}}\left(p_{1},p_{2}\right)-\left(\sqrt{{\mathrm{JS}}_{G}}\left(p_{1},p_{3}\right)+\sqrt{{\mathrm{JS}}_{G}}\left(p_{3},p_{2}\right)\right)\approx 0.1898531\ldots .$$

*Similarly, the triangle inequality also fails for the extended G-JSD: we have $\sqrt{{\mathrm{JS}}_{G}^{+}\left(p_{1},p_{2}\right)}\approx 1.0788275\ldots$, $\sqrt{{\mathrm{JS}}_{G}^{+}\left(p_{1},p_{3}\right)}\approx 0.6691922\ldots$, and $\sqrt{{\mathrm{JS}}_{G}^{+}\left(p_{3},p_{2}\right)}\approx 0.1984633\ldots$ with a triangle inequality defect value of*

$$\sqrt{{\mathrm{JS}}_{G}^{+}\left(p_{1},p_{2}\right)}-\left(\sqrt{{\mathrm{JS}}_{G}^{+}\left(p_{1},p_{3}\right)}+\sqrt{{\mathrm{JS}}_{G}^{+}\left(p_{3},p_{2}\right)}\right)\approx 0.2111719\ldots .$$

### 2.2. Power JSDs and (Extended) Min-JSD and Max-JSD

Let $P_{\gamma ,\alpha }\left(a,b\right):={\left(\alpha a^{\gamma }+\left(1-\alpha \right)b^{\gamma }\right)}^{\frac{1}{\gamma }}$ be the γ-power mean for γ≠0 (with $A_{\alpha }=P_{1,\alpha }$). Further define $P_{0,\alpha }\left(a,b\right)=G_{\alpha }\left(a,b\right)$ so that $P_{\gamma ,\alpha }$ defines the weighted power means for γ∈R and α∈(0,1) in the reminder. Since $P_{\gamma ,\alpha }\left(a,b\right)\leq P_{\gamma^{\prime },\alpha }\left(a,b\right)$ when $\gamma^{\prime }\geq \gamma$ for any a,b>0, we have

(18) $$Z_{\alpha }^{P_{\gamma }}\left(p_{1},p_{2}\right)=\int P_{\gamma ,\alpha }\left(p_{1}\left(x\right),p_{2}\left(x\right)\right)\;d\mu \leq Z_{\alpha }^{P_{\gamma^{\prime }}}\left(p_{1},p_{2}\right)=\int P_{\gamma^{\prime },\alpha }\left(p_{1}\left(x\right),p_{2}\left(x\right)\right)\;d\mu .$$

Let $P_{\gamma }\left(a,b\right)=P_{\gamma ,\frac{1}{2}}\left(a,b\right)$. We have $\lim_{\gamma \to -\infty }P_{\gamma }\left(a,b\right)=\min(a,b)$ and $\lim_{\gamma \to +\infty }P_{\gamma }\left(a,b\right)=\max(a,b)$. Thus, we can define both (extended) min-JSD and (extended) max-JSD. Using the fact that $\min(a,b)=\frac{a+b}{2}-\frac{1}{2}\left|a-b\right|$ and $\max(a,b)=\frac{a+b}{2}+\frac{1}{2}\left|a-b\right|$, we obtain the extremal mixture normalization terms as follows:

(19) $$\begin{array}{ccc} Z_{\min}\left(p_{1},p_{2}\right) & = & \int \min(p_{1},p_{2})\;d\mu =1-\mathrm{TV}(p_{1},p_{2}), \end{array}$$

(20) $$\begin{array}{ccc} Z_{\max}\left(p_{1},p_{2}\right) & = & \int \max(p_{1},p_{2})\;d\mu =1+\mathrm{TV}(p_{1},p_{2}), \end{array}$$

where $\mathrm{TV}(p_{1},p_{2})=\frac{1}{2}\int \left|p_{1}-p_{2}\right|\;d\mu$ is the total variation distance.

**Proposition** **7** (max-JSD)**.** *The following upper bound holds for *max*-JSD:*

(21) $$0\leq {{\mathrm{JS}}^{+}}_{\tilde{\max}}\left(p_{1},p_{2}\right)\leq \mathrm{TV}\left(p_{1},p_{2}\right).$$

*Furthermore, the following identity relates the two types of *max*-JSDs:*

(22) $${{\mathrm{JS}}^{+}}_{\tilde{\max}}\left(p_{1},p_{2}\right)={\mathrm{JS}}_{\tilde{\max}}\left(p_{1},p_{2}\right)+\mathrm{TV}\left(p_{1},p_{2}\right)-\log\left(1+\mathrm{TV}(p_{1},p_{2})\right).$$

**Proof.**  We have

$${{\mathrm{JS}}^{+}}_{\tilde{\max}}(p_{1},p_{2}):=\frac{1}{2}\;\int \left(p_{1}\log\frac{p_{1}}{\max(p_{1},p_{2})}+p_{2}\log\frac{p_{2}}{\max(p_{1},p_{2})}+2\max\left(p_{1},p_{2}\right)-\left(p_{1}+p_{2}\right)\right)\;d\mu .$$

Since both $\log\frac{p_{1}}{\max(p_{1},p_{2})}\leq 0$ and $\log\frac{p_{2}}{\max(p_{1},p_{2})}\leq 0$, and $\max(a,b)=\frac{a+b}{2}+\frac{1}{2}\left|b-a\right|$, we have

$${{\mathrm{JS}}^{+}}_{\tilde{\max}}\left(p_{1},p_{2}\right)\leq \int \left(\frac{p_{1}+p_{2}}{2}+\frac{1}{2}\left|p_{2}-p_{1}\right|-\frac{p_{1}+p_{2}}{2}\right)\;d\mu .$$

That is, ${{\mathrm{JS}}^{+}}_{\tilde{\max}}\left(p_{1},p_{2}\right)\leq \mathrm{TV}\left(p_{1},p_{2}\right)$.

We characterize the gap as follows:

$$\begin{array}{ccc} \Delta_{\max}\left(p_{1},p_{2}\right) & = & Z_{\max}\left(p_{1},p_{2}\right)-\log Z_{\max}\left(p_{1},p_{2}\right)-1,\\ & = & \mathrm{TV}\left(p_{1},p_{2}\right)-\log\left(1+\mathrm{TV}\left(p_{1},p_{2}\right)\right)\geq 0, \end{array}$$

since 0≤TV≤1. Thus ${{\mathrm{JS}}^{+}}_{\tilde{\max}}\left(p_{1},p_{2}\right)\geq {\mathrm{JS}}_{\max}\left(p_{1},p_{2}\right)$. □

**Proposition** **8** (min-JSD)**.** *We have the following lower bound on the extended min-JSD:*

$${{\mathrm{JS}}^{+}}_{\tilde{\min}}\left(p_{1},p_{2}\right)\geq \frac{1}{4}\;J\left(p_{1},p_{2}\right)-\mathrm{TV}\left(p_{1},p_{2}\right),$$

*where $J\left(p_{1},p_{2}\right):=\mathrm{KL}\left(p_{1},p_{2}\right)+\mathrm{KL}\left(p_{2},p_{1}\right)=\int \left(p_{1}-p_{2}\right)\log\frac{p_{1}}{p_{2}}\;d\mu$ is the Jeffreys’ divergence [2] and*

$${{\mathrm{JS}}^{+}}_{\tilde{\min}}\left(p_{1},p_{2}\right)={\mathrm{JS}}_{\min}\left(p_{1},p_{2}\right)-\mathrm{TV}\left(p_{1},p_{2}\right)+\log\left(1-\mathrm{TV}\left(p_{1},p_{2}\right)\right).$$

**Proof.**  We have $Z_{\min}\left(p_{1},p_{2}\right)=\int \min\{p_{1},p_{2}\}\;d\mu =1-\mathrm{TV}\left(p_{1},p_{2}\right)\leq 1$ and

$$\begin{array}{ccc} \Delta_{\min}\left(p_{1},p_{2}\right) & = & Z_{\min}\left(p_{1},p_{2}\right)-\log Z_{\min}\left(p_{1},p_{2}\right)-1,\\ & = & -\mathrm{TV}\left(p_{1},p_{2}\right)-\log\left(1-\mathrm{TV}\left(p_{1},p_{2}\right)\right)\geq 0, \end{array}$$

since −x−log(1−x)≥0 for x≤1. Note that the gap can be arbitrarily large when $\mathrm{TV}\left(p_{1},p_{2}\right)\to 1^{-}$.

Thus, we have ${{\mathrm{JS}}^{+}}_{\tilde{\min}}\left(p_{1},p_{2}\right)\geq {\mathrm{JS}}_{\min}\left(p_{1},p_{2}\right)$.

To get the lower bound, we use the fact that $\min(p_{1},p_{2})\leq \sqrt{p_{1}p_{2}}$. Indeed, we have

$$\begin{array}{ccc} {{\mathrm{JS}}^{+}}_{\tilde{\min}}\left(p_{1},p_{2}\right) & = & \frac{1}{2}\left(\int (p_{1}\log\frac{p_{1}}{\min(p_{1},p_{2})}+p_{2}\log\frac{p_{2}}{\min(p_{1},p_{2})}+2\min\left(p_{1},p_{2}\right)-\left(p_{1}+p_{2}\right)\right)\;d\mu ,\\ & \geq & \frac{1}{2}\int \left(\frac{1}{2}p_{1}\log\frac{p_{1}}{p_{2}}+\frac{1}{2}p_{2}\log\frac{p_{2}}{p_{1}}+2\min\left(p_{1},p_{2}\right)-\left(p_{1}+p_{2}\right)\right)\;d\mu ,\\ & = & \frac{1}{4}\;J\left(p_{1},p_{2}\right)-\mathrm{TV}\left(p_{1},p_{2}\right). \end{array}$$

□

**Remark** **9.**  *Let us report the total variation distance between two univariate Gaussian distributions $p_{\mu_{1},\sigma_{1}}$ and $p_{\mu_{2},\sigma_{2}}$ in closed form using the error function [42] $\mathrm{erf}\left(x\right)=\frac{1}{\sqrt{\pi }}\int_{-x}^{x}e^{-t^{2}}dt$.*

- *When $\sigma_{1}=\sigma_{2}=\sigma$, we have*
(23) $$\mathrm{TV}\left(p_{1},p_{2}\right)=\frac{1}{2}\left|\Phi \left(x^{*};\mu_{2},\sigma \right)-\Phi \left(x^{*};\mu_{1},\sigma \right)\right|,$$
  *where $\Phi \left(x;\mu ,\sigma \right)=\frac{1}{2}\left(1+\mathrm{erf}\left(\frac{x-\mu }{\sigma \sqrt{2}}\right)\right)$ is the cumulative distribution, and*
(24) $$x^{*}=\frac{\mu_{1}^{2}-\mu_{2}^{2}}{2(\mu_{1}-\mu_{2})}.$$
- *When $\sigma_{1}\neq \sigma_{2}$, we let $x_{1}=\frac{-b-\sqrt{\Delta }}{2a}$ and $x_{2}=\frac{-b+\sqrt{\Delta }}{2a}$, where $\Delta =b^{2}-4ac\geq 0$ and*
(25) $$\begin{array}{ccc} a & = & \frac{1}{\sigma_{1}^{2}}-\frac{1}{\sigma_{2}^{2}}, \end{array}$$
(26) $$\begin{array}{ccc} b & = & 2\left(\frac{\mu_{2}}{\sigma_{2}}-\frac{\mu_{1}}{\sigma_{1}}\right), \end{array}$$
(27) $$\begin{array}{ccc} c & = & {\left(\frac{\mu_{1}}{\sigma_{1}}\right)}^{2}-{\left(\frac{\mu_{2}}{\sigma_{2}}\right)}^{2}-2\log\frac{\sigma_{2}}{\sigma_{1}}. \end{array}$$
*The total variation is given by*
(28) $$\begin{array}{ccc} \mathrm{TV}(p_{1},p_{2})=\\ & & \frac{1}{2}\left(\left|\mathrm{erf}\left(\frac{x_{1}-\mu_{1}}{\sigma_{1}\sqrt{2}}\right)-\mathrm{erf}\left(\frac{x_{1}-\mu_{2}}{\sigma_{2}\sqrt{2}}\right)\right|+\left|\mathrm{erf}\left(\frac{x_{2}-\mu_{1}}{\sigma_{1}\sqrt{2}}\right)-\mathrm{erf}\left(\frac{x_{2}-\mu_{2}}{\sigma_{2}\sqrt{2}}\right)\right|\right) \end{array}$$

Next, we shall consider the important case of $p_{1}$ and $p_{2}$ belonging to the family of multivariate normal distributions, commonly called Gaussian distributions.

## 3. Geometric JSDs Between Gaussian Distributions

### 3.1. Exponential Families

The formula for the G-JSD between two Gaussian distributions was reported in [17] using the more general framework of exponential families. An exponential family [43] is a family of probability measures $\left\{P_{\lambda }\right\}$ with Radon–Nikodym densities $p_{\lambda }$ with respect to μ expressed canonically as

$$\begin{array}{ccc} p_{\lambda }\left(x\right) & := & \exp\left(\langle \theta (\lambda ),t(x)\rangle -F\left(\theta \right)+k\left(x\right)\right),\\ & = & \frac{1}{Z(\theta )}\;\exp\left(\langle \theta (\lambda ),t(x)\rangle +k\left(x\right)\right), \end{array}$$

where θ(λ) is the natural parameter, t(x) the sufficient statistic, k(x) an auxiliary carrier term with respect to μ, and F(θ) the cumulant function. The partition function Z(θ) is the normalizer denominator: Z(θ)=exp(F(θ)). The cumulant function F(θ)=logZ(θ) is strictly convex and analytic [43], and the partition function Z(θ)=exp(F(θ)) is strictly log-convex (and hence also strictly convex).

We consider the exponential family of multivariate Gaussian distributions

$$N=\{N\left(\mu ,\Sigma \right)\;:\;\mu \in R^{d},\Sigma \in \mathrm{PD}\left(d\right)\},$$

where PD(d) denotes the set of symmetric positive–definite matrices of size d×d. Let $\lambda :=\left(\lambda_{v},\lambda_{M}\right)=\left(\mu ,\Sigma \right)$ denote the compound (vector, matrix) parameter of a Gaussian. The *d*-variate Gaussian density is given by

(29) $$\begin{array}{ccc} p_{\lambda }\left(x;\lambda \right) & := & \frac{1}{{\left(2\pi \right)}^{\frac{d}{2}}\sqrt{|\lambda_{M}|}}\exp\left(-\frac{1}{2}{\left(x-\lambda_{v}\right)}^{⊤}\lambda_{M}^{-1}\left(x-\lambda_{v}\right)\right), \end{array}$$

where |·| denotes the matrix determinant. The natural parameters θ are expressed using both a vector parameter $\theta_{v}$ and a matrix parameter $\theta_{M}$ in a compound parameter $\theta =(\theta_{v},\theta_{M})$. By defining the following compound inner product on a compound (vector, matrix) parameter

(30) $$\langle \theta ,\theta^{\prime }\rangle :=\theta_{v}^{⊤}\theta_{v}^{\prime }+\mathrm{tr}\left({\theta_{M}^{\prime }}^{⊤}\theta_{M}\right),$$

where tr(·) denotes the matrix trace, we rewrite the Gaussian density of Equation (29) in the canonical form of an exponential family:

(31) $$\begin{array}{ccc} p_{\theta }\left(x;\theta \right) & := & \exp\left(\langle t(x),\theta \rangle -F_{\theta }\left(\theta \right)\right)=p_{\lambda }\left(x\right), \end{array}$$

where θ=θ(λ) with

(32) $$\theta =\left(\theta_{v},\theta_{M}\right)=\left(\Sigma^{-1}\mu ,-\frac{1}{2}\Sigma^{-1}\right)=\theta \left(\lambda \right)=\left(\lambda_{M}^{-1}\lambda_{v},-\frac{1}{2}\lambda_{M}^{-1}\right),$$

is the compound vector-matrix natural parameter and

(33) $$t\left(x\right)=\left(x,-xx^{⊤}\right),$$

is the compound vector-matrix sufficient statistic. There is no auxiliary carrier term (i.e., k(x)=0). The function $F_{\theta }$ is given by

(34) $$F_{\theta }\left(\theta \right):=\frac{1}{2}\left(d\log\pi -\log|\theta_{M}|+\frac{1}{2}\theta_{v}^{⊤}\theta_{M}^{-1}\theta_{v}\right),$$

**Remark** **10.**  *Beware that when the cumulant function is expressed using the ordinary parameter λ=(μ,Σ), the cumulant function $F_{\theta }\left(\theta \left(\lambda \right)\right)$ is no longer convex:*

(35) $$\begin{array}{ccc} F_{\lambda }\left(\lambda \right) & = & \frac{1}{2}\left(\lambda_{v}^{⊤}\lambda_{M}^{-1}\lambda_{v}+\log\left|\lambda_{M}\right|+d\log2\pi \right), \end{array}$$

(36) $$\begin{array}{ccc} & = & \frac{1}{2}\left(\mu^{⊤}\Sigma^{-1}\mu +\log\left|\Sigma \right|+d\log2\pi \right). \end{array}$$

We convert between the ordinary parameterization λ=(μ,Σ) and the natural parameterization θ using these formulas:

$$\begin{array}{ccc} \theta =\left(\theta_{v},\theta_{M}\right)=\left\{\begin{array}{c} \theta_{v}\left(\lambda \right)=\lambda_{M}^{-1}\lambda_{v}=\Sigma^{-1}\mu \\ \theta_{M}\left(\lambda \right)=\frac{1}{2}\lambda_{M}^{-1}=\frac{1}{2}\Sigma^{-1} \end{array}\right. & \Leftrightarrow & \lambda =\left(\lambda_{v},\lambda_{M}\right)=\left\{\begin{array}{c} \lambda_{v}\left(\theta \right)=\frac{1}{2}\theta_{M}^{-1}\theta_{v}=\mu \\ \lambda_{M}\left(\theta \right)=\frac{1}{2}\theta_{M}^{-1}=\Sigma \end{array}\right. \end{array}$$

The geometric mixture $p_{\theta_{1}}^{\alpha }p_{\theta_{2}}^{1-\alpha }$ of two densities of an exponential family is a density $p_{\alpha \theta_{1}+\left(1-\alpha \right)\theta_{2}}$ of the exponential family with the partition function $Z_{\alpha }\left(\theta_{1},\theta_{2}\right)=\exp\left(-J_{F,\alpha }\left(\theta_{1},\theta_{2}\right)\right)$, where $J_{F,\alpha }\left(\theta_{1},\theta_{2}\right)$ denotes the skew Jensen divergence [44,45]:

$$J_{F,\alpha }\left(\theta_{1},\theta_{2}\right):=\alpha F\left(\theta_{1}\right)+\left(1-\alpha \right)F\left(\theta_{2}\right)-F\left(\alpha \theta_{1}+\left(1-\alpha \right)\theta_{2}\right).$$

Therefore, the difference gap of Equation (17) between the G-JSD and the extended G-JSD between exponential family densities is given by

(37) $$\begin{array}{ccc} \Delta (\theta_{1},\theta_{2}) & = & \exp\left(-J_{F,\alpha }\left(\theta_{1},\theta_{2}\right)\right)+J_{F,\alpha }\left(\theta_{1},\theta_{2}\right)-1, \end{array}$$

(38) $$\begin{array}{ccc} & = & Z_{\alpha }\left(\theta_{1},\theta_{2}\right)-\log Z_{\alpha }\left(\theta_{1},\theta_{2}\right)-1, \end{array}$$

(39) $$\begin{array}{ccc} & = & Z_{\alpha }\left(\theta_{1},\theta_{2}\right)-F\left(\alpha \theta_{1}+\left(1-\alpha \right)\theta_{2}\right)-1. \end{array}$$

Since $Z_{\alpha }=\exp\left(-J_{F,\alpha }\left(\theta_{1},\theta_{2}\right)\right)\leq 1$, the gap Δ is negative, and we have

$${{\mathrm{JS}}^{+}}_{{\tilde{G}}_{\alpha },\beta }\left(p_{\mu_{1},\Sigma_{1}},p_{\mu_{2},\Sigma_{2}}\right)\leq {\mathrm{JS}}_{G_{\alpha },\beta }\left(p_{\mu_{1},\Sigma_{1}},p_{\mu_{2},\Sigma_{2}}\right).$$

**Corollary** **2.**  *When $p_{1}=p_{\theta_{1}}$ and $p_{2}=p_{\theta_{2}}$ belongs to a same exponential family with the cumulant function F(θ), we have*

(40) $${\mathrm{JS}}_{G}\left(p_{\theta_{1}},p_{\theta_{2}}\right)=\frac{1}{4}{\left(\theta_{2}-\theta_{1}\right)}^{⊤}\left(\nabla F\left(\theta_{2}\right)-\nabla F\left(\theta_{1}\right)\right)-\left(\frac{F\left(\theta_{1}\right)+F\left(\theta_{2}\right)}{2}-F\left(\frac{\theta_{1}+\theta_{2}}{2}\right)\right),$$

*since $J\left(p_{\theta_{1}},p_{\theta_{2}}\right)=\langle \theta_{2}-\theta_{1},\nabla F\left(\theta_{2}\right)-\nabla F\left(\theta_{1}\right)\rangle$ amounts to a symmetrized Bregman divergence.*

**Proof.**  We have $J\left(p_{\theta_{1}},p_{\theta_{2}}\right)={\left(\theta_{2}-\theta_{1}\right)}^{⊤}\left(\nabla F\left(\theta_{2}\right)-\nabla F\left(\theta_{1}\right)\right)$ and $J\left(p_{\theta_{1}},p_{\theta_{2}}\right)=J_{F}\left(\theta_{1},\theta_{2}\right)$. □

The extended geometric Jensen–Shannon divergence and geometric Jensen–Shannon divergence between two densities of an exponential family are given by

$$\begin{array}{ccc} {\mathrm{JS}}_{G}\left(p_{\theta_{1}},p_{\theta_{2}}\right) & = & \frac{1}{4}{\left(\theta_{2}-\theta_{1}\right)}^{⊤}\left(\nabla F\left(\theta_{2}\right)-\nabla F\left(\theta_{1}\right)\right)-\left(\frac{F\left(\theta_{1}\right)+F\left(\theta_{2}\right)}{2}-F\left(\frac{\theta_{1}+\theta_{2}}{2}\right)\right),\\ {\mathrm{JS}}_{\tilde{G}}\left(p_{\theta_{1}},p_{\theta_{2}}\right) & = & \frac{1}{4}\langle \theta_{2}-\theta_{1},\nabla F\left(\theta_{2}\right)-\nabla F\left(\theta_{1}\right)\rangle -\exp\left(-J_{F}\left(\theta_{1},\theta_{2}\right)\right)-1,\\ {{\mathrm{JS}}^{*}}_{G}\left(p_{\theta_{1}},p_{\theta_{2}}\right) & = & J_{F}\left(\theta_{1},\theta_{2}\right) \end{array}$$

**Remark** **11.**  *Given two densities $p_{1}$ and $p_{2}$, the family G of geometric mixtures $\left\{{\left(p_{1}p_{2}\right)}_{G_{\alpha }}\propto p_{1}^{\alpha }\;p_{2}^{1-\alpha }\;:\;\alpha \in \left(0,1\right)\right\}$ forms a 1D exponential family that has been termed the likelihood ratio exponential family [46] (LREF). The cumulant function of this LREF is $F\left(\alpha \right)=-B_{\alpha }\left(p_{1},p_{2}\right)$. Hence, G has also been called a Bhattacharyya arc or Hellinger arc in the literature [47]. However, notice that $\mathrm{KL}(p_{i}:{\left(p_{1}p_{2}\right)}_{G_{\alpha }})$ does not necessarily amount to a Bregman divergence, because neither $p_{1}$ nor $p_{2}$ belongs to G.*

### 3.2. Closed-Form Formula for Gaussian Distributions

Let us report the corresponding closed-form formula for *d*-variate Gaussian distributions.

When $\alpha =\frac{1}{2}$, we proved that ${\mathrm{JS}}_{G}\left(p_{1},p_{2}\right)=\frac{1}{4}\;J\left(p_{1},p_{2}\right)-B\left(p_{1},p_{2}\right)$ and ${\mathrm{JS}}_{\tilde{G}}^{+}\left(p_{1},p_{2}\right)=\frac{1}{4}\;J\left(p_{1},p_{2}\right)+\exp\left(-B\left(p_{1},p_{2}\right)\right)-1$ where $\mathrm{BC}\left(p_{1},p_{2}\right)=\exp\left(-B\left(p_{1},p_{2}\right)\right)$. Thus, for the case of balanced geometric mixtures, we need to report the closed form for the Jeffreys and Bhattacharyya distances:

$$\begin{array}{ccc} J(p_{\mu_{1},\Sigma_{1}},p_{\mu_{2},\Sigma_{2}}) & = & \frac{1}{2}\;\left(\mathrm{tr}\left(\Sigma_{1}\Sigma_{2}^{-1}+\Sigma_{2}\Sigma_{1}^{-1}\right)+{\left(\mu_{1}-\mu_{2}\right)}^{⊤}\left(\Sigma_{1}^{-1}+\Sigma_{2}^{-1}\right)\left(\mu_{1}-\mu_{2}\right)-2d\right)\;,\\ B(p_{\mu_{1},\Sigma_{1}},p_{\mu_{2},\Sigma_{2}}) & = & \frac{1}{8}\;{\left(\mu_{1}-\mu_{2}\right)}^{⊤}{\bar{\Sigma }}^{-1}\left(\mu_{1}-\mu_{2}\right)+\frac{1}{2}\log\left(\frac{\det\bar{\Sigma }}{\sqrt{\det\Sigma_{1}\;\det\Sigma_{2}}}\right), \end{array}$$

where $\bar{\Sigma }=\frac{1}{2}\;\left(\Sigma_{1}+\Sigma_{2}\right)$.

Otherwise, for the arbitrary weighted geometric mixture $G_{\alpha }$, define ${\left(\theta_{1}\theta_{2}\right)}_{\alpha }=\alpha \theta_{1}+\left(1-\alpha \right)\theta_{2}$, the weighted linear interpolation of the natural parameters $\theta_{1}$ and $\theta_{2}$.

**Corollary** **3.**  The skew G-Jensen–Shannon divergence ${\mathrm{JS}}_{\alpha }^{G}$ and the dual skew G-Jensen–Shannon divergence ${{\mathrm{JS}}^{*}}_{\alpha }^{G}$ between two d-variate Gaussian distributions $N(\mu_{1},\Sigma_{1})$ and $N(\mu_{2},\Sigma_{2})$ is

$$\begin{array}{ccc} {\mathrm{JS}}_{G_{\alpha }}\left(p_{\left(\mu_{1},\Sigma_{1}\right)},p_{\left(\mu_{2},\Sigma_{2}\right)}\right) & = & \alpha \;\mathrm{KL}\left(p_{\left(\mu_{1},\Sigma_{1}\right)},p_{\left(\mu_{\alpha },\Sigma_{\alpha }\right)}\right)+\left(1-\alpha \right)\;\mathrm{KL}\left(p_{\left(\mu_{2},\Sigma_{2}\right)},p_{\left(\mu_{\alpha },\Sigma_{\alpha }\right)}\right),\\ & = & \alpha \;B_{F}\left({\left(\theta_{1}\theta_{2}\right)}_{\alpha },\theta_{1}\right)+\left(1-\alpha \right)\;B_{F}\left({\left(\theta_{1}\theta_{2}\right)}_{\alpha },\theta_{2}\right),\\ & = & \frac{1}{2}\left(\mathrm{tr}\left(\Sigma_{\alpha }^{-1}\left(\alpha \Sigma_{1}+\left(1-\alpha \right)\Sigma_{2}\right)\right)+\log\left(\frac{|\Sigma_{\alpha }|}{|\Sigma_{1}{|}^{\alpha }{\left|\Sigma_{2}\right|}^{1-\alpha }}\right)\right.\\ & = & \left.+\alpha {\left(\mu_{\alpha }-\mu_{1}\right)}^{⊤}\Sigma_{\alpha }^{-1}\left(\mu_{\alpha }-\mu_{1}\right)+\left(1-\alpha \right){\left(\mu_{\alpha }-\mu_{2}\right)}^{⊤}\Sigma_{\alpha }^{-1}\left(\mu_{\alpha }-\mu_{2}\right)-d\right)\\ {\mathrm{JS}}_{G_{\alpha }}^{*}\left(p_{\left(\mu_{1},\Sigma_{1}\right)},p_{\left(\mu_{2},\Sigma_{2}\right)}\right) & = & \left(1-\alpha \right)\;\mathrm{KL}\left(p_{\left(\mu_{\alpha },\Sigma_{\alpha }\right)},p_{\left(\mu_{1},\Sigma_{1}\right)}\right)+\alpha \mathrm{KL}\left(p_{\left(\mu_{\alpha },\Sigma_{\alpha }\right)},p_{\left(\mu_{2},\Sigma_{2}\right)}\right),\\ & = & \alpha \;B_{F}\left(\theta_{1},{\left(\theta_{1}\theta_{2}\right)}_{\alpha }\right)+\left(1-\alpha \right)\;B_{F}\left(\theta_{2},{\left(\theta_{1}\theta_{2}\right)}_{\alpha }\right),\\ & = & J_{F,\alpha }\left(\theta_{1},\theta_{2}\right)=:B_{\alpha }\left(p_{\left(\mu_{1},\Sigma_{1}\right)},p_{\left(\mu_{2},\Sigma_{2}\right)}\right),\\ & = & \frac{1}{2}\left(\alpha \mu_{1}^{⊤}\Sigma_{1}^{-1}\mu_{1}+\left(1-\alpha \right)\mu_{2}^{⊤}\Sigma_{2}^{-1}\mu_{2}-\mu_{\alpha }^{⊤}\Sigma_{\alpha }^{-1}\mu_{\alpha }+\log\frac{|\Sigma_{1}{|}^{\alpha }{\left|\Sigma_{2}\right|}^{1-\alpha }}{|\Sigma_{\alpha }|}\right),\\ F(\mu ,\Sigma ) & = & \frac{1}{2}\left(\mu^{⊤}\Sigma^{-1}\mu +\log\left|\Sigma \right|+d\log2\pi \right),\\ F(\theta_{v},\theta_{M}) & = & \frac{1}{2}\left(d\log\pi -\log|\theta_{M}|+\frac{1}{2}\theta_{v}^{⊤}\theta_{M}^{-1}\theta_{v}\right),\\ \Delta (\theta_{1},\theta_{2}) & = & \exp\left(-J_{F,\alpha }\left(\theta_{1},\theta_{2}\right)\right)+J_{F,\alpha }\left(\theta_{1},\theta_{2}\right)-1, \end{array}$$

*where $\Sigma_{\alpha }$ is the matrix harmonic barycenter:*

(41) $$\Sigma_{\alpha }={\left(\alpha \Sigma_{1}^{-1}+\left(1-\alpha \right)\Sigma_{2}^{-1}\right)}^{-1},$$

*and*

(42) $$\mu_{\alpha }=\Sigma_{\alpha }\left(\alpha \Sigma_{1}^{-1}\mu_{1}+\left(1-\alpha \right)\Sigma_{2}^{-1}\mu_{2}\right).$$

## 4. The Extended and Normalized G-JSDs as Regularizations of the Ordinary JSD

The *M*-Jensen–Shannon divergence ${\mathrm{JS}}_{M}\left(p,q\right)$ can be interpreted as a regularization of the ordinary JSD:

**Proposition** **9** (JSD regularization)**.** *For any arbitrary mean M, the following identity holds:*

(43) $${\mathrm{JS}}_{M}\left(p_{1},p_{2}\right)=\mathrm{JS}\left(p_{1},p_{2}\right)+\mathrm{KL}\left(\frac{p_{1}+p_{2}}{2},{\left(p_{1}p_{2}\right)}_{M}\right).$$

Notice that ${\left(p_{1}p_{2}\right)}_{A}=\frac{p_{1}+p_{2}}{2}$.

**Proof.**  We have

$$\begin{array}{ccc} {\mathrm{JS}}_{M}\left(p_{1},p_{2}\right) & := & \frac{1}{2}\left(\mathrm{KL}\left(p_{1},{\left(p_{1}p_{2}\right)}_{M}\right)+\mathrm{KL}\left(p_{2},{\left(p_{1}p_{2}\right)}_{M}\right)\right),\\ & = & \frac{1}{2}\int \left(p_{1}\log\frac{p_{1}\;{\left(p_{1}p_{2}\right)}_{A}}{{\left(p_{1}p_{2}\right)}_{M}\;{\left(p_{1}p_{2}\right)}_{A}}+p_{2}\log\frac{p_{2}\;{\left(p_{1}p_{2}\right)}_{A}}{{\left(p_{1}p_{2}\right)}_{M}\;{\left(p_{1}p_{2}\right)}_{A}}\right)\;d\mu ,\\ & = & \frac{1}{2}\int \left(p_{1}\log\frac{p_{1}}{{\left(p_{1}p_{2}\right)}_{A}}+p_{1}\log\frac{{\left(p_{1}p_{2}\right)}_{A}}{{\left(p_{1}p_{2}\right)}_{M}}+p_{2}\log\frac{p_{2}}{{\left(p_{1}p_{2}\right)}_{A}}+p_{2}\log\frac{{\left(p_{1}p_{2}\right)}_{A}}{{\left(p_{1}p_{2}\right)}_{M}}\right)\;d\mu ,\\ & = & \frac{1}{2}\int \left(p_{1}\log\frac{p_{1}}{{\left(p_{1}p_{2}\right)}_{A}}+p_{2}\log\frac{p_{2}}{{\left(p_{1}p_{2}\right)}_{A}}\right)\;d\mu +\int \frac{1}{2}\;\left(p_{1}+p_{2}\right)\log\frac{{\left(p_{1}p_{2}\right)}_{A}}{{\left(p_{1}p_{2}\right)}_{M}}\;d\mu ,\\ & = & \mathrm{JS}\left(p_{1},p_{2}\right)+\int {\left(p_{1}p_{2}\right)}_{A}\log\frac{{\left(p_{1}p_{2}\right)}_{A}}{{\left(p_{1}p_{2}\right)}_{M}}\;d\mu ,\\ & = & \mathrm{JS}\left(p_{1},p_{2}\right)+\mathrm{KL}\left({\left(p_{1}p_{2}\right)}_{A},{\left(p_{1}p_{2}\right)}_{M}\right). \end{array}$$

□

**Remark** **12.**  *One way to symmetrize the KLD is to consider two distinct symmetric means $M_{1}\left(a,b\right)=M_{1}\left(b,a\right)$ and $M_{2}\left(a,b\right)=M_{2}\left(b,a\right)$ and define*

$${\mathrm{KL}}_{M_{1},M_{2}}\left(p_{1},p_{2}\right)=\mathrm{KL}\left({\left(p_{1}p_{2}\right)}_{M_{1}},{\left(p_{1}p_{2}\right)}_{M_{2}}\right)={\mathrm{KL}}_{M_{1},M_{2}}\left(p_{2},p_{1}\right).$$

*We notice that $\sqrt{{\mathrm{KL}}^{A,G}}$ is not a metric distance by reporting a triple of points $\left(p_{1},p_{2},p_{3}\right)$ that fails the triangle inequality. Consider $p_{1}=\left(0.55,0.45\right)$, $p_{2}=\left(0.002,0.998\right)$, and $p_{3}=\left(0.045,0.955\right)$. We have $\sqrt{{\mathrm{KL}}_{M_{1},M_{2}}\left(p_{1},p_{2}\right)}=0.5374165\ldots$, $\sqrt{{\mathrm{KL}}_{M_{1},M_{2}}\left(p_{1},p_{3}\right)}=0.1759400\ldots$, and $\sqrt{{\mathrm{KL}}_{M_{1},M_{2}}\left(p_{3},p_{2}\right)}=0.08485931\ldots$. The triangle inequality defect is*

$$\sqrt{{\mathrm{KL}}_{M_{1},M_{2}}\left(p_{1},p_{2}\right)}-\left(\sqrt{{\mathrm{KL}}_{M_{1},M_{2}}\left(p_{1},p_{3}\right)}+\sqrt{{\mathrm{KL}}_{M_{1},M_{2}}\left(p_{3},p_{2}\right)}\right)=0.2766171\ldots .$$

*We can also similarly symmetrize the extended KLD as follows:*

$${\mathrm{KL}}_{{\tilde{M}}_{1},{\tilde{M}}_{2}}^{+}\left(q_{1},q_{2}\right)={\mathrm{KL}}^{+}\left({\left(q_{1}q_{2}\right)}_{{\tilde{M}}_{1}},{\left(q_{1}q_{2}\right)}_{{\tilde{M}}_{2}}\right)={\mathrm{KL}}_{{\tilde{M}}_{1},{\tilde{M}}_{2}}\left(q_{2},q_{1}\right).$$

*In particular, when $M_{1}=A$ and $M_{2}=G$, we get the ${\mathrm{KL}}_{A,M}$ divergence:*

$${\mathrm{KL}}_{A,M}\left(p_{1},p_{2}\right)=\frac{p_{1}+p_{2}}{2}\;\log\frac{p_{1}+p_{2}}{2\sqrt{p_{1}p_{2}}}+\log Z_{G}\left(p_{1},p_{2}\right),$$

*which is related to Taneja T-divergence [48]:*

(44) $$T\left(p_{1},p_{2}\right)=\int \frac{p_{1}+p_{2}}{2}\log\frac{p_{1}+p_{2}}{2\sqrt{p_{1}p_{2}}}.$$

*The T-divergence is an f-divergence [11,12] obtained for the generator $f_{T}\left(u\right)=\frac{1+u}{2}\log\frac{1+u}{2\sqrt{u}}$.*

**Corollary** **4** (JSD lower bound on *M*-JSD)**.** *We have ${\mathrm{JS}}_{M}\left(p,q\right)\geq \mathrm{JS}\left(p,q\right)$.*

**Proof.**  Since ${\mathrm{JS}}_{M}\left(p,q\right)=\mathrm{JS}\left(p,q\right)+\mathrm{KL}\left(\frac{p+q}{2},{\left(pq\right)}_{M}\right)$ and KL≥0 by Gibbs’ inequality, we have ${\mathrm{JS}}_{M}\left(p,q\right)\geq \mathrm{JS}\left(p,q\right)$. □

Since the extended *M*-JSD is ${\mathrm{JS}}_{{\tilde{M}}_{\alpha },\beta }^{+}\left(p_{1},p_{2}\right)={\mathrm{JS}}_{M_{\alpha },\beta }\left(p_{1},p_{2}\right)+Z_{\alpha }-\log\left(Z_{\alpha }\right)-1$, the extended *M*-JSD ${\mathrm{JS}}_{{\tilde{M}}_{\alpha },\beta }^{+}$ can also be interpreted as another regularization of the Jensen–Shannon divergence when dealing with probability densities:

(45) $${\mathrm{JS}}_{{\tilde{M}}_{\alpha },\beta }^{+}\left(p_{1},p_{2}\right)=\mathrm{JS}\left(p_{1},p_{2}\right)+\mathrm{KL}\left(\frac{p_{1}+p_{2}}{2},{\left(p_{1}p_{2}\right)}_{M}\right)+Z_{M_{\alpha }}\left(p_{1},p_{2}\right)-\log\left(Z_{M_{\alpha }}\left(p_{1},p_{2}\right)\right)-1.$$

It is well known that the JSD can be rewritten as a diversity index [4] using concave entropy:

(46) $$\mathrm{JS}\left(p_{1},p_{2}\right)=H\left(\frac{p_{1}+p_{2}}{2}\right)-\frac{H\left(p_{1}\right)+H\left(p_{2}\right)}{2}.$$

We generalize this decomposition as the difference of a cross-entropy term minus entropies, as follows:

**Proposition** **10** (M-JSD cross-entropy decomposition)**.** *We have*

$${\mathrm{JS}}_{M}\left(p_{1},p_{2}\right)=H^{\times }\left({\left(p_{1}p_{2}\right)}_{A},{\left(p_{1}p_{2}\right)}_{M}\right)-\frac{H\left(p_{1}\right)+H\left(p_{2}\right)}{2}.$$

**Proof.**  From Proposition 9, we have

$${\mathrm{JS}}_{M}\left(p_{1},p_{2}\right)=\mathrm{JS}\left(p_{1},p_{2}\right)+\mathrm{KL}\left(\frac{p_{1}+p_{2}}{2},{\left(p_{1}p_{2}\right)}_{M}\right).$$

Since $\mathrm{KL}\left(p_{1},p_{2}\right)=H^{\times }\left(p_{1},p_{2}\right)-H\left(p_{1}\right)$, where $H^{\times }\left(p_{1},p_{2}\right)=-\int p_{1}\log p_{2}\;d\mu$ is the cross-entropy and $H\left(p\right)=-\int p\log p\;d\mu =H^{\times }\left(p,p\right)$ is the entropy. Plugging Equation (46) into Equation (43), we get

$$\begin{array}{ccc} {\mathrm{JS}}_{M}\left(p_{1},p_{2}\right) & = & H\left(\frac{p_{1}+p_{2}}{2}\right)-\frac{H\left(p_{1}\right)+H\left(p_{2}\right)}{2}+H^{\times }\left(\frac{p_{1}+p_{2}}{2},{\left(p_{1}p_{2}\right)}_{M}\right)-H\left(\frac{p_{1}+p_{2}}{2}\right),\\ & = & H^{\times }\left(\frac{p_{1}+p_{2}}{2},{\left(p_{1}p_{2}\right)}_{M}\right)-\frac{H\left(p_{1}\right)+H\left(p_{2}\right)}{2}. \end{array}$$

Note that when M=A, the arithmetic mean, we have $H^{\times }\left(\frac{p_{1}+p_{2}}{2},{\left(p_{1}p_{2}\right)}_{M}\right)=H\left(\frac{p_{1}+p_{2}}{2}\right)$ and we recover the fact that ${\mathrm{JS}}_{M}\left(p_{1},p_{2}\right)=\mathrm{JS}\left(p_{1},p_{2}\right)$. □

## 5. Estimation and Approximation of the Extended and Normalized M-JSDs

Let us recall the two definitions of the extended M-JSD and the normalized M-JSD (for the case of $\alpha =\beta =\frac{1}{2}$) between two normalized densities $p_{1}$ and $p_{2}$:

$$\begin{array}{ccc} {\mathrm{JS}}_{M}\left(p_{1},p_{2}\right) & = & \frac{1}{2}\left(\mathrm{KL}\left(p_{1},{\left(p_{1}p_{2}\right)}_{M}\right)+\mathrm{KL}\left(p_{2},{\left(p_{1}p_{2}\right)}_{M}\right)\right),\\ {\mathrm{JS}}_{M}^{+}\left(p_{1},p_{2}\right) & = & \frac{1}{2}\left({\mathrm{KL}}^{+}\left(p_{1},{\left(p_{1}p_{2}\right)}_{\tilde{M}}\right)+{\mathrm{KL}}^{+}\left(p_{2},{\left(p_{1}p_{2}\right)}_{\tilde{M}}\right)\right), \end{array}$$

where ${\left(p_{1}p_{2}\right)}_{M}\left(x\right)=\frac{M(p_{1}\left(x\right),p_{2}\left(x\right))}{Z_{M}\left(p_{1},p_{2}\right)}$ (with $Z_{M}\left(p_{1},p_{2}\right)=\int M\left(p_{1}\left(x\right),p_{2}\left(x\right)\right)\;d\mu \left(x\right)$) and ${\left(p_{1}p_{2}\right)}_{\tilde{M}}\left(x\right)=M\left(p_{1}\left(x\right),p_{2}\left(x\right)\right)$.

In practice, one needs to estimate the extended and normalized G-JSDs when they do not admit a closed-form formula.

### 5.1. Monte Carlo Estimators

To estimate ${\mathrm{JS}}_{M}\left(p_{1},p_{2}\right)$, we can use Monte Carlo samplings to estimate both KLD integrals and mixture normalizers $Z_{M}$; for example, the normalizer $Z_{M}\left(p_{1},p_{2}\right)$ is estimated by

$${\hat{Z}}_{M}\left(p_{1},p_{2}\right)=\frac{1}{s}\sum_{i=1}^{s}\frac{1}{r(x_{i})}\;M\left(p_{1}\left(x_{i}\right),p_{2}\left(x_{i}\right)\right),$$

where r(x) is the proposal distribution which can be chosen according to the mean *M* and the types of probability distributions $p_{1}$ and $p_{2}$, and $x_{1},\ldots ,x_{s}$ are *s* identically and independently sampled (iid.) from r(x). However, since ${\left(p_{1}p_{2}\right)}_{M}\left(x\right)$ is now estimated as ${\left(p_{1}p_{2}\right)}_{\hat{M}\left(x\right)}$, it is no longer a normalized *M*-mixture, and thus we consider estimating

$${\mathrm{JS}}_{\hat{M}}^{+}\left(p_{1},p_{2}\right)=\frac{1}{2}\left({\mathrm{KL}}^{+}\left(p_{1},{\left(p_{1}p_{2}\right)}_{\hat{M}}\right)+{\mathrm{KL}}^{+}\left(p_{2},{\left(p_{1}p_{2}\right)}_{\hat{M}}\right)\right)$$

to ensure the non-negativity of the divergence ${\mathrm{JSD}}_{\hat{M}}$.

Let us consider the estimation of the term

$${\mathrm{KL}}^{+}\left(p_{1},{\left(p_{1}p_{2}\right)}_{\tilde{M}}\right)=\int \left(p_{1}\log\frac{p_{1}}{M(p_{1},p_{2})}+M\left(p_{1},p_{2}\right)-p_{1}\right)\;d\mu .$$

By choosing the proposal distribution $r\left(x\right)=p_{1}\left(x\right)$, we have ${\mathrm{KL}}^{+}\left(p_{1},{\left(p_{1}p_{2}\right)}_{\hat{M}}\right)\approx \hat{{\mathrm{KL}}^{+}}\left(p_{1},{\left(p_{1}p_{2}\right)}_{\tilde{M}}\right)$ (for large enough *s*), where

$${\hat{\mathrm{KL}}}^{+}\left(p_{1},{\left(p_{1}p_{2}\right)}_{\tilde{M}}\right)=\frac{1}{s}\sum_{i=1}^{s}\left(\log\frac{p_{1}\left(x_{i}\right)}{M(p_{1}\left(x_{i}\right),p_{2}\left(x_{i}\right))}+\frac{1}{p_{1}\left(x_{i}\right)}\;M\left(p_{1}\left(x_{i}\right),p_{2}\left(x_{i}\right)\right)-1\right).$$

Monte Carlo (MC) stochastic integration [49] is a well-studied topic in statistics, with many results available regarding the consistency and variance of MC estimators.

Note that even if we have a generic formula for the G-JSD between two densities of an exponential family given by Corollary 2, the cumulant function F(θ) may not be in closed form [50,51]. This is the case when the sufficient statistic vector of the exponential family is $t\left(x\right)=\left(x,x^{2},\ldots ,x^{m}\right)$ (for m≥5), yielding the polynomial exponential family (also called exponential–polynomial family [51]).

### 5.2. Approximations via γ-Divergences

One way to circumvent the lack of computational tractable density normalizers is to consider the family of γ-divergences [39] instead of the KLD:

$${\tilde{D}}_{\gamma }\left(q_{1},q_{2}\right)=\frac{1}{\gamma (1+\gamma )}\log I_{\gamma }\left(q_{1},q_{2}\right)-\frac{1}{\gamma }\log I_{\gamma }\left(q_{1},q_{2}\right)+\frac{1}{1+\gamma }\log I_{\gamma }\left(q_{1},q_{2}\right),\;\gamma >0,$$

where

$$I_{\gamma }\left(q_{1},q_{2}\right)=\int q_{1}\left(x\right)\;q_{2}^{\gamma }\left(x\right)\;d\mu \left(x\right).$$

The γ-divergences are projective divergences, i.e., they enjoy the property that

$${\tilde{D}}_{\gamma }\left(\lambda_{1}q_{1},\lambda_{2}q_{2}\right)={\tilde{D}}_{\gamma }\left(q_{1},q_{2}\right),\;\forall \lambda_{1}>0,\;\lambda_{2}>0.$$

Furthermore, we have $\lim_{\gamma \to 0}{\tilde{D}}_{\gamma }\left(p_{1},p_{2}\right)=\mathrm{KL}\left(p_{1},p_{2}\right)$. (Note that the KLD is not projective.)

Let us define the projective *M*-JSD:

(47) $${\mathrm{JS}}_{\tilde{M},\gamma }\left(p_{1},p_{2}\right)=\frac{1}{2}\left({\tilde{D}}_{\gamma }\left(p_{1},{\left(p_{1}p_{2}\right)}_{\tilde{M}}\right)+{\tilde{D}}_{\gamma }\left(p_{2},{\left(p_{1}p_{2}\right)}_{\tilde{M}}\right)\right).$$

We have, for γ=ϵ, a small enough value (e.g., $\epsilon \leq 10^{-3}$), ${\mathrm{JS}}_{M}\left(p_{1},p_{2}\right)\approx {\mathrm{JS}}_{\tilde{M},\gamma }\left(p_{1},p_{2}\right)$, since

$$\mathrm{KL}\left(p_{1},{\left(p_{1}p_{2}\right)}_{M}\right)\approx_{\gamma =\epsilon }{\tilde{D}}_{\gamma }\left(p_{1},{\left(p_{1}p_{2}\right)}_{\tilde{M}}\right).$$

In particular, for exponential family densities $p_{\theta_{1}}\left(x\right)=\frac{q_{\theta_{1}}\left(x\right)}{Z(\theta_{1})}$ and $p_{\theta_{2}}\left(x\right)=\frac{q_{\theta_{2}}\left(x\right)}{Z(\theta_{2})}$, we have

$$I_{\gamma }\left(p_{\theta_{1}},p_{\theta_{2}}\right)=\exp\left(F\left(\theta_{1}+\gamma \theta_{2}\right)-F\left(\theta_{1}\right)-\gamma F\left(\theta_{2}\right)\right),$$

provided that $\theta_{1}+\gamma \theta_{2}$ belongs to the natural parameter space (otherwise, the integral $I_{\gamma }$ diverges).

Even when F(θ) is not known in closed form, we may estimate the γ-divergence by estimating the $I_{\gamma }$ integrals as follows:

$${\hat{I}}_{\gamma }\left(q_{\theta_{1}},q_{\theta_{2}}\right)\approx \frac{1}{s}\sum_{i=1}^{s}q_{2}\left(x_{i}\right),$$

where $x_{1},\ldots ,x_{s}$ are iid. sampled from $p_{1}\left(x\right)$. For example, we may use Monte Carlo importance sampling methods [52] or exponential family Langevin dynamics [53] to sample densities of exponential family densities with computationally intractable normalizers (e.g., polynomial exponential families).

## 6. Summary and Concluding Remarks

In this paper, we first recalled the Jensen–Shannon symmetrization (JS-symmetrization) scheme of [17] for an arbitrary statistical dissimilarity D(·,·) using an arbitrary weighted scalar mean $M_{\alpha }$ as follows:

$$D_{M_{\alpha },\beta }^{\mathrm{JS}}\left(p_{1},p_{2}\right):=\beta \;D\left(p_{1},{\left(p_{1}p_{2}\right)}_{M_{\alpha }}\right)+\left(1-\beta \right)\;D\left(p_{2},{\left(p_{1}p_{2}\right)}_{M_{\alpha }}\right),\;\left(\alpha ,\beta \right)\in {\left(0,1\right)}^{2},$$

In particular, we showed that the skewed Bhattacharyya distance and the Chernoff information can both be interpreted as JS-symmetrizations of the reverse Kullback–Leibler divergence.

Then we defined two types of geometric Jensen–Shannon divergence between probability densities. The first type ${\mathrm{JS}}_{M}$ requires normalization of *M*-mixtures and relies on the Kullback–Leibler divergence: ${\mathrm{JS}}_{M}={\mathrm{KL}}_{M_{\frac{1}{2}},\frac{1}{2}}^{\mathrm{JS}}$. The second type ${\mathrm{JS}}_{\tilde{M}}^{+}$ does not normalize *M*-mixtures and uses the extended Kullback–Leibler divergence ${\mathrm{KL}}^{+}$ to take into account unnormalized mixtures: ${\mathrm{JS}}_{\tilde{M}}^{+}={\mathrm{KL}}_{{\tilde{M}}_{\frac{1}{2}},\frac{1}{2}}^{{\mathrm{JS}}^{+}}$. When *M* is the arithmetic mean *A*, both *M*-JSD types coincide with the ordinary Jensen–Shannon divergence of Equation (2).

We have shown that both *M*-JSD types can be interpreted as regularized Jensen–Shannon divergences JS with additive terms. Namely, we have

$$\begin{array}{ccc} {\mathrm{JS}}_{M}\left(p_{1},p_{2}\right) & = & \mathrm{JS}(p_{1},p_{2})+\mathrm{KL}\left({\left(p_{1}p_{2}\right)}_{A},{\left(p_{1}p_{2}\right)}_{M}\right),\\ {\mathrm{JS}}_{\tilde{M}}^{+}\left(p_{1},p_{2}\right) & = & {\mathrm{JS}}_{M}\left(p_{1},p_{2}\right)+Z_{M}\left(p_{1},p_{2}\right)-\log Z_{M}\left(p_{1},p_{2}\right)-1,\\ & = & \mathrm{JS}(p_{1},p_{2})+\mathrm{KL}\left({\left(p_{1}p_{2}\right)}_{A},{\left(p_{1}p_{2}\right)}_{M}\right)+Z_{M}\left(p_{1},p_{2}\right)-\log Z_{M}\left(p_{1},p_{2}\right)-1, \end{array}$$

where $Z_{M}\left(p_{1},p_{2}\right)=\int M\left(p_{1},p_{2}\right)\;d\mu$ is the *M*-mixture normalizer. The gap between these two types of M-JSD is

$$\begin{array}{ccc} \Delta_{M}\left(p_{1},p_{2}\right) & = & {\mathrm{JS}}_{\tilde{M}}^{+}\left(p_{1},p_{2}\right)-{\mathrm{JS}}_{M}\left(p_{1},p_{2}\right),\\ & = & Z_{M}\left(p_{1},p_{2}\right)-\log Z_{M}\left(p_{1},p_{2}\right)-1. \end{array}$$

When taking the geometric mean M=G, we showed that both G-JSD types can be expressed using the Jeffreys divergence and the Bhattacharyya divergence (or Bhattacharyya coefficient):

$$\begin{array}{ccc} {\mathrm{JS}}_{G}\left(p_{1},p_{2}\right) & = & \frac{1}{4}\;J\left(p_{1},p_{2}\right)-B\left(p_{1},p_{2}\right),\\ {\mathrm{JS}}_{\tilde{G}}^{+}\left(p_{1},p_{2}\right) & = & \frac{1}{4}\;J\left(p_{1},p_{2}\right)+\exp\left(-B\left(p_{1},p_{2}\right)\right)-1,\\ & = & \frac{1}{4}\;J\left(p_{1},p_{2}\right)+\mathrm{BC}\left(p_{1},p_{2}\right)-1. \end{array}$$

Thus, the gap between these two types of G-JSD is

$$\begin{array}{ccc} \Delta_{G}\left(p_{1},p_{2}\right) & := & {\mathrm{JS}}_{\tilde{G}}^{+}\left(p_{1},p_{2}\right)-{\mathrm{JS}}_{G}\left(p_{1},p_{2}\right),\\ & = & \mathrm{BC}\left(p_{1},p_{2}\right)+B\left(p_{1},p_{2}\right)-1,\\ & = & Z_{G}\left(p_{1},p_{2}\right)-\log Z_{G}\left(p_{1},p_{2}\right)-1, \end{array}$$

since $Z_{G}\left(p_{1},p_{2}\right)=\int \sqrt{p_{1}\;p_{2}}d\mu =\mathrm{BC}\left(p_{1},p_{2}\right)$.

Although the square root of the Jensen–Shannon divergence yields a metric distance, this is no longer the case for the geometric-JSD and the extended geometric-JSD: we reported counterexamples in Remark 8. Moreover, we have shown that the KL symmetrization $\sqrt{\mathrm{KL}({\left(p_{1}p_{2}\right)}_{A},{\left(p_{1}p_{2}\right)}_{G})}$ is not a metric distance (Remark 12).

We discussed the merit of the extended G-JSD, which does not require normalization of the geometric mixture, in Section 5, and we showed how to approximate the G-JSD using the projective γ-divergences [39] for γ=ϵ, a small enough value (i.e., $\gamma =\epsilon =10^{-3}$). From the viewpoint of information geometry, the extended G-JSD has been shown to be an *f*-divergence [13] (separable divergence), while the G-JSD is not separable in general because of the normalization of mixtures (with the exception of the ordinary JSD, which is an *f*-divergence because the arithmetic mixtures do not require normalization).

We studied power JSDs by considering the power means and studied the ±∞ limits, the extended max-JSD, and the min-JSD: We proved that the extended max-JSD is upper-bounded by the total variation distance $\mathrm{TV}\left(p_{1},p_{2}\right)=\frac{1}{2}\;\int \left|p_{1}-p_{2}\right|\;d\mu$:

$$0\leq {\mathrm{JS}}_{\tilde{\max}}^{+}\left(p_{1},p_{2}\right)\leq \mathrm{TV}\left(p_{1},p_{2}\right),$$

and that the extended min-JSD is lower-bounded as follows:

$${\mathrm{JS}}_{\tilde{\min}}^{+}\left(p_{1},p_{2}\right)\geq \frac{1}{4}\;J\left(p_{1},p_{2}\right)-\mathrm{TV}\left(p_{1},p_{2}\right),$$

where *J* denotes the Jeffreys divergence: $J\left(p_{1},p_{2}\right)=\mathrm{KL}\left(p_{1},p_{2}\right)+\mathrm{KL}\left(p_{2},p_{1}\right)$.

The advantage of using the extended G-JSD is that we do not need to normalize geometric mixtures, while this novel divergence is proven to be an *f*-divergence [13] and retains the property that it amounts to a regularization of the ordinary Jensen–Shannon divergence by an extra additive gap term.

Finally, we expressed ${\mathrm{JS}}_{G}$ (Equation (41)) and ${\mathrm{JS}}_{\tilde{G}}^{+}$ (Equation (41)) for exponential families, characterized the gap between these two types of divergences as a function of the cumulant and partition functions, and reported a corresponding explicit formula for the multivariate Gaussian (exponential) family. The G-JSD between Gaussian distributions has already been used successfully in many applications [30,32,33,34,35,36,37,38].

## Data Availability

No new data were created or analyzed in this study. Data sharing is not applicable to this article.

## Nomenclature

Means:	
$M_{\alpha }\left(a,b\right)$	weighted scalar mean
$M_{\alpha }^{\phi }\left(a,b\right)$	weighted quasi-arithmetic scalar mean for generator ϕ(u)
A(a,b)	arithmetic mean
$A_{\alpha }\left(a,b\right)$	weighted arithmetic mean
$G_{\alpha }\left(a,b\right)$	weighted geometric mean
G(a,b)	geometric mean
$P_{\gamma }\left(a,b\right)$	power mean with $P_{0}=G$ and $P_{1}=A$
$P_{\gamma ,\alpha }\left(a,b\right)$	weighted power mean
Densities on measure space (X,E,μ):
$p,p_{1},p_{2},\ldots$	normalized density
$q,q_{1},q_{2},\ldots$	unnormalized density
Z(q)	density normalizer $p=\frac{q}{Z(q)}$
$Z_{M}\left(p_{1},p_{2}\right)$	normalizer of *M*-mixture ($\alpha =\frac{1}{2}$)
${\hat{Z}}_{M}\left(p_{1},p_{2}\right)$	Monte Carlo estimator of $Z_{M}\left(p_{1},p_{2}\right)$
$Z_{M,\alpha }\left(p_{1},p_{2}\right)$	normalizer of weighted *M*-mixture
${\left(p_{1}p_{2}\right)}_{M}$	*M*-mixture
${\left(p_{1}p_{2}\right)}_{M,\alpha }$	weighted *M*-mixture
Dissimilarities, divergences, and distances:
$\mathrm{KL}(p_{1},p_{2})$	Kullback–Leibler divergence (KLD)
${\mathrm{KL}}^{+}\left(q_{1},q_{2}\right)$	extended Kullback–Leibler divergence
${\mathrm{KL}}^{*}\left(p_{1},p_{2}\right)$	reverse Kullback–Leibler divergence
$H^{\times }\left(p_{1},p_{2}\right)$	cross-entropy
H(p)	Shannon discrete or differential entropy
$J(p_{1},p_{2})$	Jeffreys divergence
$\mathrm{TV}(p_{1},p_{2})$	total variation distance
$B(p_{1},p_{2})$	Bhattacharyya “distance” (not metric)
$B_{\alpha }\left(p_{1},p_{2}\right)$	α-skewed Bhattacharyya “distance”
$C(p_{1},p_{2})$	Chernoff information or Chernoff distance
$T(p_{1},p_{2})$	Taneja *T*-divergence
$I_{f}\left(p_{1},p_{2}\right)$	Ali–Silvey–Csiszár *f*-divergence
$D(p_{1},p_{2})$	arbitrary dissimilarity measure
$D^{*}\left(p_{1},p_{2}\right)$	reverse dissimilarity measure
$D^{+}\left(q_{1},q_{2}\right)$	extended dissimilarity measure
$\tilde{D}\left(q_{1},q_{2}\right)$	projective dissimilarity measure
${\tilde{D}}_{\gamma }\left(q_{1},q_{2}\right)$	γ-divergence
${\hat{D}}^{+}\left(q_{1},q_{2}\right)$	Monte Carlo estimation of dissimilarity $D^{+}$
Jensen–Shannon divergences and generalizations:
$\mathrm{JS}(p_{1},p_{2})$	Jensen–Shannon divergence (JSD)
${\mathrm{JS}}_{\alpha ,\beta }\left(p_{1},p_{2}\right)$	β-weighted α-skewed mixture JSD
${\mathrm{JS}}_{M}\left(p_{1},p_{2}\right)$	*M*-JSD for *M*-mixtures
${\mathrm{JS}}_{G}\left(p_{1},p_{2}\right)$	geometric JSD
${\mathrm{JS}}_{\tilde{G}}\left(p_{1},p_{2}\right)$	extended geometric JSD
${\mathrm{JS}}_{G}^{*}\left(p_{1},p_{2}\right)$	left-sided geometric JSD (right-sided for ${\mathrm{KL}}^{*}$)
${{\mathrm{JS}}^{+}}_{\tilde{\min}}\left(p_{1},p_{2}\right)$	min-JSD
${{\mathrm{JS}}^{+}}_{\tilde{\max}}\left(p_{1},p_{2}\right)$	max-JSD
$\Delta_{M}\left(p_{1},p_{2}\right)$	gap between extended and normalized M-JSDs
